# A longitudinal single-cell atlas of anti-tumour necrosis factor treatment in inflammatory bowel disease

**DOI:** 10.1038/s41590-024-01994-8

**Published:** 2024-10-22

**Authors:** Tom Thomas, Matthias Friedrich, Charlotte Rich-Griffin, Mathilde Pohin, Devika Agarwal, Julia Pakpoor, Carl Lee, Ruchi Tandon, Aniko Rendek, Dominik Aschenbrenner, Ashwin Jainarayanan, Alexandru Voda, Jacqueline H. Y. Siu, Raphael Sanches-Peres, Eloise Nee, Dharshan Sathananthan, Dylan Kotliar, Peter Todd, Maria Kiourlappou, Lisa Gartner, Nicholas Ilott, Fadi Issa, Joanna Hester, Jason Turner, Saba Nayar, Jonas Mackerodt, Fan Zhang, Anna Jonsson, Michael Brenner, Soumya Raychaudhuri, Ruth Kulicke, Danielle Ramsdell, Nicolas Stransky, Ray Pagliarini, Piotr Bielecki, Noah Spies, Brian Marsden, Stephen Taylor, Allon Wagner, Paul Klenerman, Alissa Walsh, Mark Coles, Luke Jostins-Dean, Fiona M. Powrie, Andrew Filer, Simon Travis, Holm H. Uhlig, Calliope A. Dendrou, Christopher D. Buckley

**Affiliations:** 1 Kennedy Institute of Rheumatology, University of Oxford, Oxford, UK; 2 Centre for Human Genetics, University of Oxford, Oxford, UK; 3 Translational Gastroenterology & Liver Unit, John Radcliffe Hospital, Headington, Oxford, UK; 4 University College London Hospitals NHS Foundation Trust, London, UK; 5 Oxford University Hospitals NHS Foundation Trust, Oxford, UK; 6 University of Adelaide, Adelaide, Australia; 7 Lyell McEwin Hospital, Adelaide, Australia; 8 Broad Institute of MIT and Harvard, Cambridge, MA USA; 9 Department of Medicine, Brigham and Women’s Hospital, Boston, MA USA; 10 Nuffield Department of Surgical Sciences, University of Oxford, Oxford, UK; 11 Rheumatology Research Group, Institute of Inflammation and Ageing, University of Birmingham, Birmingham, UK; 12 National Institute for Health Research (NIHR) Birmingham Biomedical Research Centre and NIHR Clinical Research Facility, University Hospitals Birmingham NHS Foundation Trust, Birmingham, UK; 13 Birmingham Tissue Analytics, Institute of Translational Medicine, University of Birmingham, Birmingham, UK; 14 Center for Health AI, University of Colorado Anschutz, Anschutz, CO USA; 15 Celsius Therapeutics, Cambridge, MA USA; 16 Department of Electrical Engineering and Computer Science, University of California, Berkeley, Berkeley, CA USA; 17 The Center for Computational Biology, University of California, Berkeley, Berkeley, CA USA; 18 NIHR Oxford Biomedical Research Centre, Oxford, UK; 19 Department of Paediatrics, University of Oxford, Oxford, UK

**Keywords:** Crohn's disease, Autoinflammatory syndrome

## Abstract

Precision medicine in immune-mediated inflammatory diseases (IMIDs) requires a cellular understanding of treatment response. We describe a therapeutic atlas for Crohn’s disease (CD) and ulcerative colitis (UC) following adalimumab, an anti-tumour necrosis factor (anti-TNF) treatment. We generated ~1 million single-cell transcriptomes, organised into 109 cell states, from 216 gut biopsies (41 subjects), revealing disease-specific differences. A systems biology-spatial analysis identified granuloma signatures in CD and interferon (IFN)-response signatures localising to T cell aggregates and epithelial damage in CD and UC. Pretreatment differences in epithelial and myeloid compartments were associated with remission outcomes in both diseases. Longitudinal comparisons demonstrated disease progression in nonremission: myeloid and T cell perturbations in CD and increased multi-cellular IFN signalling in UC. IFN signalling was also observed in rheumatoid arthritis (RA) synovium with a lymphoid pathotype. Our therapeutic atlas represents the largest cellular census of perturbation with the most common biologic treatment, anti-TNF, across multiple inflammatory diseases.

## Abstract (web summary)

In this Resource, Buckley and colleagues profile patients with Crohn’s disease and ulcerative colitis before and after adalimumab therapy. Specific pretreatment differences in the epithelial and myeloid compartments were associated with remission outcomes in both diseases. The authors also describe the cellular circuitry in nonremission patients following treatment.

## Main

Immune-mediated inflammatory diseases (IMIDs) are characterised by impaired immune tolerance leading to chronic inflammation and end-organ damage. The discovery that anti-TNF therapy ameliorates inflammation marked a new era in IMID treatment^1–3^. However, with nonresponse rates reaching 40% and nondurable remission, medications beyond anti-TNF are required for many patients, including those with CD, UC and RA^4–7^.

Recent studies have explored the cellular^8–20^ and molecular^21–27^ basis of these diseases and their histopathological features^28^. However, cellular distinctions between inflamed CD and UC, and their respective tissue niches, remain poorly understood. Although previous inflammatory bowel disease (IBD) studies have implicated activated fibroblasts^13,14,28^, neutrophils^26–28^, inflammatory monocytes^13,29^ and activated T and IgG^+^ plasma cells^8,13^ with anti-TNF nonresponse, no biomarker is currently approved for response prediction. As such, and given the current plethora of treatment options, formulating effective drug sequencing strategies following anti-TNF failure is an urgent clinical need. Understanding the cellular impact of therapeutic agents can inform these strategies, yet no study has directly interrogated the tissue landscape of IMIDs before and after anti-TNF in adults using single-cell RNA sequencing (scRNA-seq).

Here, we aimed to create a cellular census of CD and UC to deliver a proof-of-concept therapeutic atlas as a precision medicine resource. Through the TAURUS study, we characterised the cellular associations of disparate treatment outcomes in the context of the most commonly used biologic therapy class. We also extended our approach to the RA synovium.

## Results

### A longitudinal scRNA-seq atlas of adalimumab in CD and UC

**Fig. 1:**
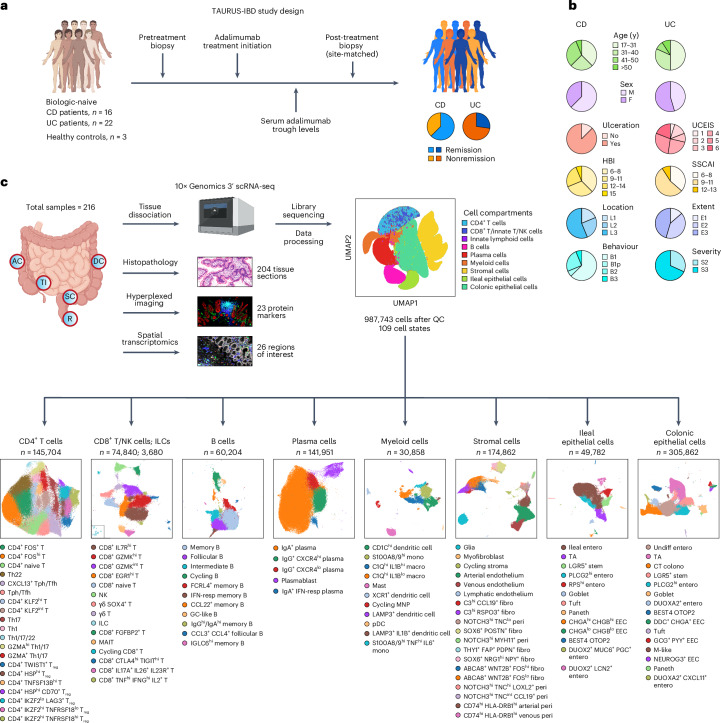
An overview of the TAURUS study. **a**, ‘Tissue biomarkers for AdalimUmab in inflammatory bowel disease and RheUmatoid arthritiS’ (TAURUS)-IBD study design outlining sample collection before and after treatment from biologic naïve patients with IBD. **b**, Clinical characteristics of patients included in TAURUS-IBD. See Supplementary Table 1 for more details. **c**, TAURUS workflow outlining number of high-quality transcriptomes (987,743 cells) generated across compartments with associated cell states and uniform manifold approximation and projection visualisations. AC, ascending colon; CD, Crohn’s disease; colono, colonocyte; DC, descending colon; EEC, enteroendocrine cell; entero, enterocyte; F, female; fibro, fibroblast; GC, germinal centre; hi, high; HBI, Harvey-Bradshaw Index; IFN-resp, interferon-responsive; ILC, innate lymphoid cell; lo, low; M, male; macro, macrophage; MAIT, mucosal-associated invariant T; MNP, mononuclear phagocyte; mono, monocyte; NK, natural killer cells; pDC, plasmacytoid dendritic cell; peri, pericyte; R, rectum; RPS^hi^, ribosomal protein S-high; SSCAI, Simple Clinical Colitis Activity Index; SC, sigmoid colon; TA, transit-amplifying; Tfh, CD4^+^ follicular helper T cell; Tph, CD4^+^ peripheral helper T cell; Th, CD4^+^ T helper cell; TI, terminal ileum; T_reg_, CD4^+^ regulatory T cell; UCEIS, Ulcerative Colitis Endoscopic Index of Severity; UC, ulcerative colitis; Undiff, undifferentiated.

**Extended Data Fig. 1:**
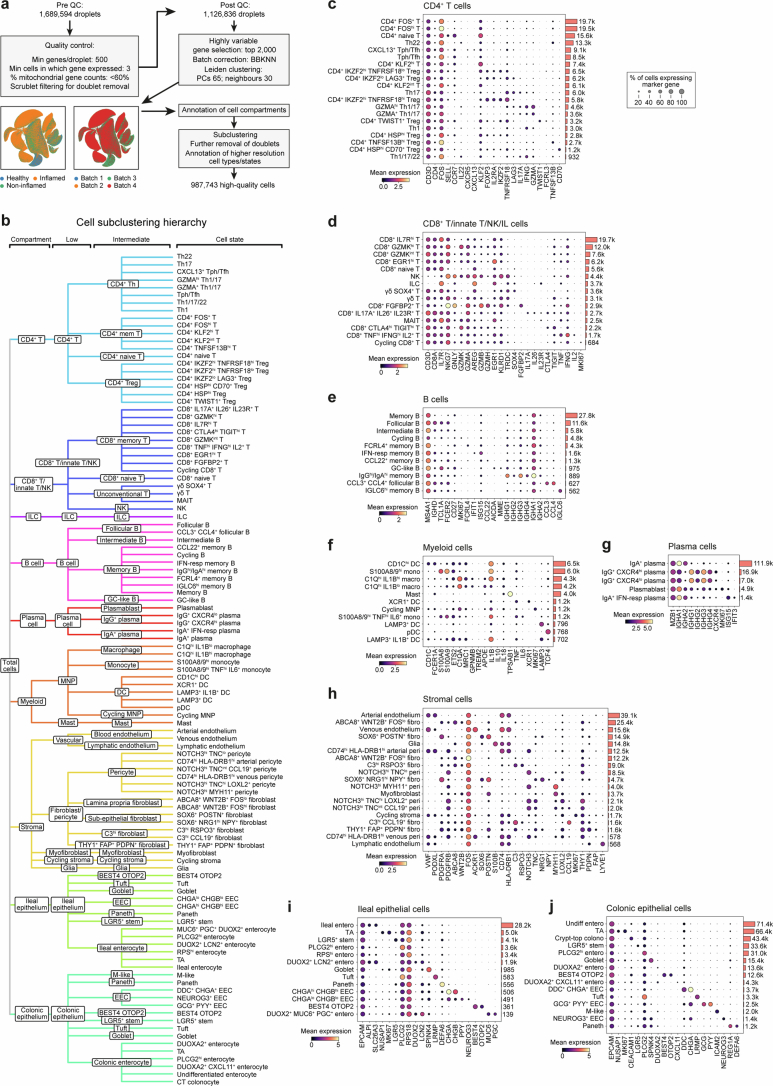
Sample processing, annotation hierarchy and gut cell state markers. **a**, Schematic showing bioinformatic pre-processing strategy for gut samples. Panpipes pipeline was used for pre-processing. Uniform manifold approximation and projection (UMAP) visualisations show the cellular landscape of gut samples coloured by inflammation status, and batch. See Methods for more details. **b**, Hierarchy shows annotation across increasing cell type resolution: compartment, low, intermediate and cell state. Dotplots showing expression of marker genes of cell states in the scRNA-seq dataset: (**c**) CD4^+^ T cell, (**d**) CD8^+^ T/innate T/NK/IL cell, (**e**) B cell, (**f**) myeloid cell, (**g**) plasma cell, (**h**) stromal cell, (**i**) ileal epithelial cell and (**j**) colonic epithelial cell. Genes relate to Supplementary Table 2. Colono, colonocyte; DC, dendritic cell; EEC, enteroendocrine cell; entero, enterocyte; fibro, fibroblast; GC, germinal centre; hi, high; IFN-resp, interferon-responsive; ILC, innate lymphoid cell; lo, low; macro, macrophage; MAIT, mucosal-associated invariant T; MNP, mononuclear phagocyte; mono, monocyte; NK, natural killer; PC: principal components; pDC, plasmacytoid dendritic cell; peri, pericyte; TA, transit-amplifying; Tfh, CD4^+^ follicular helper T cell; Th, CD4^+^ helper T cell; Tph, CD4^+^ peripheral helper T cell; Treg, regulatory T cell; Undiff, undifferentiated.

We collected biopsies from 38 biologic-naïve patients with CD or UC and three healthy controls across five gut regions (terminal ileum, ascending colon, descending colon, sigmoid and rectum) before and after treatment with adalimumab (Fig. 1a and Supplementary Table 1). Eighty-nine percent of patients (*n* = 34) had at least one pair of site-matched longitudinal biopsies. Our study comprises 987,743 high-quality single-cell transcriptomes from 216 gut samples (Fig. 1a and Extended Data Fig. 1). Subclustering of nine immune, stromal and epithelial cell compartments yielded 109 distinct cell states (Extended Data Fig. 1b–j and Supplementary Table 2).

### Epithelial heterogeneity drives mRNA variation by gut site

**Extended Data Fig. 2:**
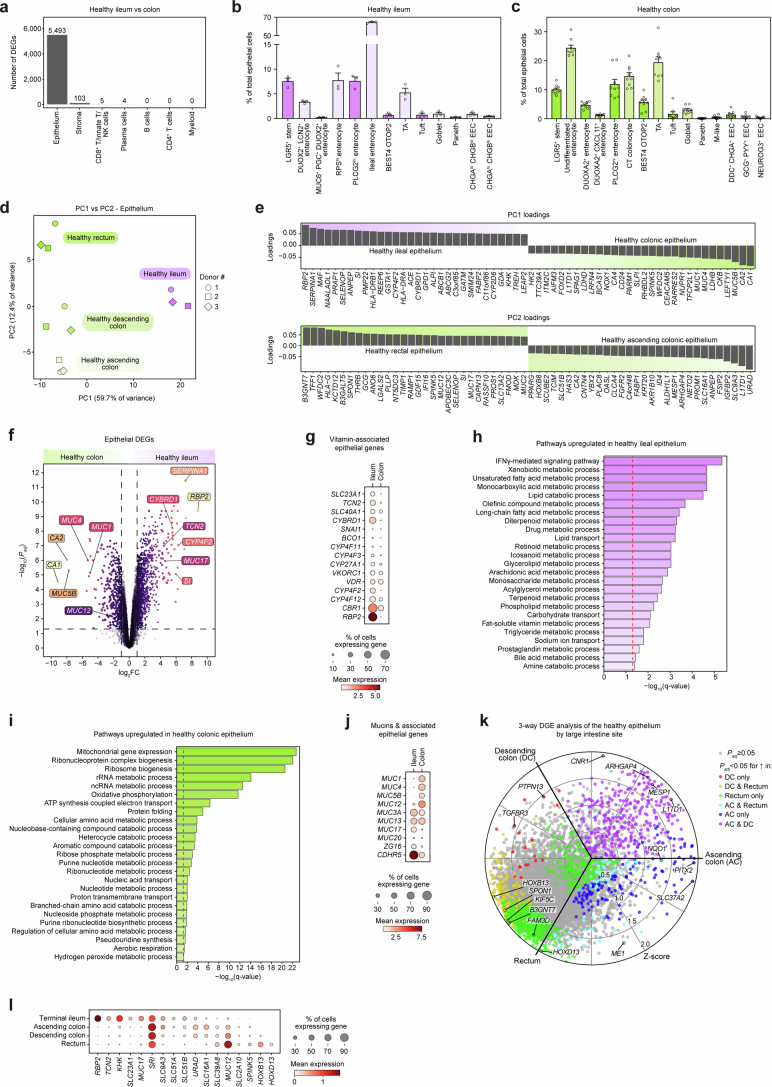
Differences between the healthy ileum and colon. **a**, Barplot summarising number of differentially expressed genes (DEGs) (*P*_*adj*_ < 0.05) comparing healthy ileum (three samples) to healthy colon (nine samples) in three patients in each cell compartment. Limma-voom with DuplicateCorrelation used to adjust for multiple samples per patient. **b,c**, Cell state distribution within the epithelial compartment in (**b**) ileum and (**c**) colon displayed on a barplot. Error bar indicates standard error of mean. Sample numbers as in (**a**). **d**, prcomp from base R used to conduct PCA on CPM normalised and log-transformed read counts. Samples in context of principal components (PC) 1 and 2 along with associated percentage of variation explained. **e**, Loadings of genes associated with PC1 and PC2 shown in the barplots. **f**, Volcano plot showing results of differential expression (limma-voom) between ileum and colon in the epithelial compartment. Dashed lines demarcate two-sided BH-corrected *P*_adj_ = 0.05 and log_2_ fold change (FC) = 0.5. **g**, Relative expression of vitamin-associated epithelial genes differentially expressed between ileum and colon shown in dotplot. Full results can be found in Supplementary Table 3. **h,i**, Overrepresentation analysis was performed by using the enrichGO function from clusterProfiler^85^. All genes significantly associated with (**h**) ileum and (**i**) colon respectively tested for overrepresentation using gene ontology (GO) biological process gene sets. Red dashed line indicative of q-value = 0.05. **j**, Relative expression of mucin and mucin-associated genes differentially expressed between ileum and colon shown in dotplot. Full results can be found in Supplementary Table 3. **k**, Three-way DGE analysis of the healthy epithelium comparing descending, ascending colon and rectum. **l**, Dotplot of key differentially expressed genes by gut site.

As variance in our transcriptomic dataset could be attributable to biopsy region, we examined healthy samples for differences between terminal ileum and colon (Extended Data Fig. 2a–c and Supplementary Table 3). Most differences were in the epithelium with 5,493 differentially expressed genes (DEGs) (Extended Data Fig. 2a). Principal component analysis (PCA) demonstrated that 59.7% of epithelial variance (PC1) was explained by ileal and colonic differences (Extended Data Fig. 2d). PC2 (12.4%) highlighted differences along the colon. Genes involved in vitamin absorption/metabolism and fatty acid metabolism were preferentially expressed in the ileum (Extended Data Fig. 2e–i). Mucin expression varied by site: *MUC17* was preferentially expressed in the ileum, whereas *MUC1*, *MUC4*, *MUC5B* and *MUC12* showed predominantly colonic expression (Extended Data Fig. 2j). Within the colon, solute carrier genes (metal ion influx and glucose transport) were enriched distally (Extended Data Fig. 2k,l).

### A molecular approach to quantifying inflammation

**Fig. 2:**
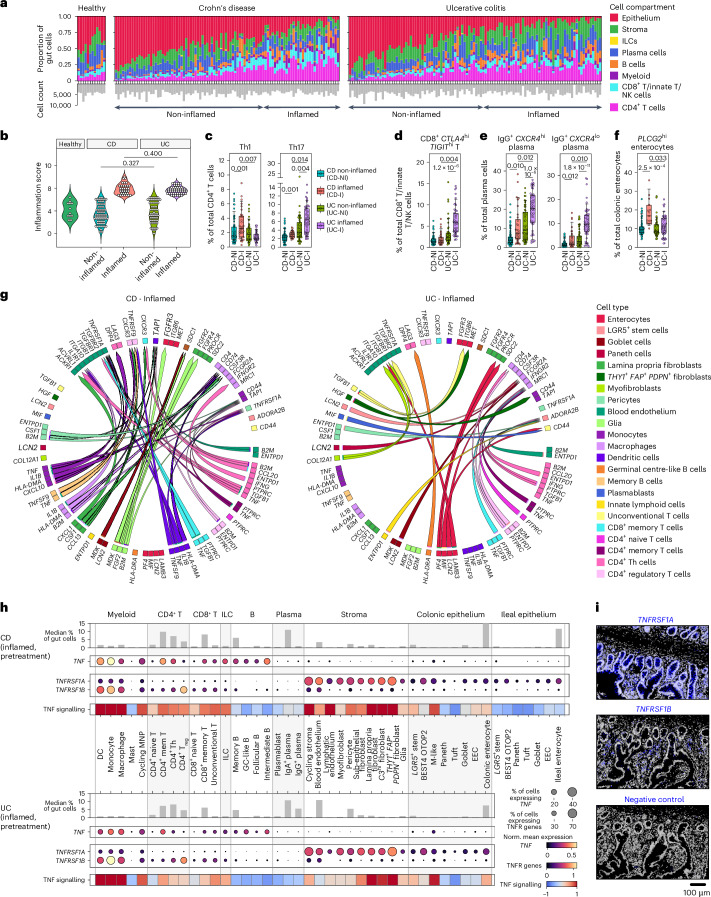
Epithelial and lymphocyte stoichiometry underpins cellular distinctions between CD and UC. **a**, Stacked barplots showing proportion of cell compartments within individual gut samples and barplot of per sample cell counts. Samples are ordered according to inflammatory score. **b**, Violin plots showing distribution of inflammation scores across healthy (*n* = 12 samples from 3 patients), CD (*n* = 33 inflamed, 63 noninflamed samples from 16 patients) and UC (*n* = 50 inflamed, 53 noninflamed samples from 22 patients) samples. Wilcoxon rank-sum test used to test significance (two-sided). **c–f**, Boxplots showing cell state as a proportion of the ‘low’ resolution cell subpopulations (see Extended Data Fig. 1 for cellular hierarchy), for CD noninflamed (CD-NI), CD inflamed (CD-I), UC noninflamed (UC-NI) and UC inflamed (UC-I) gut samples. Boxplots show median, first (lower hinge) and third (upper hinge) quartiles; whiskers show 1.5× interquartile range. Sample numbers as in (**b**). MASC was used to test abundance across inflammation status and disease with nested random effects accounting for multiple samples per patient, and covariates (Methods). Only significant (two-sided *P*_*adj*_ < 0.05) differences after multiple comparisons correction with Benjamini-Hochberg (BH) are shown. **g**, Cell-cell interaction plots showing ligand-receptor pairs enriched in inflamed CD versus inflamed UC. **h**, Mean expression of mRNA transcripts at the ‘intermediate’ cell resolution is shown for *TNF*, *TNFRSF1A* and *TNFRSF1B* in pretreatment inflamed samples in CD and UC. PROGENy was applied to pretreatment inflamed samples to calculate TNF signalling scores^43^. Heatmap shows relative enrichment of TNF signalling scores. Barplots show median cell percentage of total cells. **i**, Spatial distribution of *TNFRSF1A* and *TNFRSF1B* in the gut compared to negative control (RNAscope); three serial sections per probe from one patient. DC, dendritic cell; EEC, enteroendocrine cell; entero, enterocyte; GC, germinal centre; hi: high; ILC, innate lymphoid cell; lo, low; MNP, mononuclear phagocyte; Th, CD4^+^ T helper cell.

**Extended Data Fig. 3:**
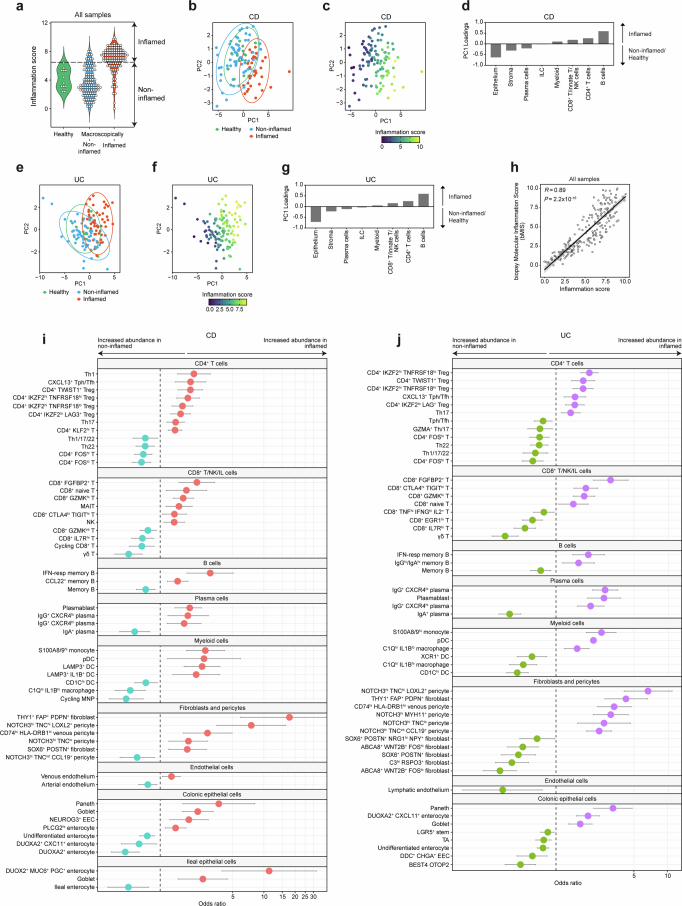
The inflammation score in context of CD and UC. **a**, Violin plot showing the distribution of the inflammation score across healthy and macroscopically noninflamed, as well as inflamed samples. **b**, PCA examining compartment abundance as a proportion of sample in CD. **c**, PCA of samples with CD with inflammation score plotted as a quantitative variable. **d**, PC1 loadings associated with cell compartment in samples with CD. **e**, PCA examining compartment abundance as a proportion of sample in UC. **f**, PCA of samples with UC with inflammation score plotted as a quantitative variable **g**, PC1 loadings associated with cell compartment in samples with UC **h**, Spearman correlation between inflammation score per sample and the recently described biopsy molecular inflammation score (bMIS). Line indicates linear regression with 95% confidence interval (grey band), two-sided *P* value shown. **i, j**, Differential abundance of cell states in CD (**i**) and UC (**j**) comparing noninflamed to inflamed tissue. Sample numbers as in Fig. 2b. Circles indicate odds ratios. Error bars show 95% confidence interval. DC, dendritic cell; hi, high; IFN-resp, interferon-responsive; ILC, innate lymphoid cell; lo, low; macro, macrophage; MAIT, mucosal-associated invariant T; MNP, mononuclear phagocyte; mono, monocyte; NK, natural killer; pDC, plasmacytoid dendritic cell; TA, transit-amplifying; Tfh, CD4^+^ follicular helper T cell; Th, CD4^+^ helper T cell; Tph, CD4^+^ peripheral helper T cell; Treg, regulatory T cell.

Previous research has highlighted that macroscopically noninflamed gut samples can be histologically and transcriptomically inflamed^14^. We generated a gene-based inflammation score using an external IBD dataset^28^. We used this score to quantify inflammation in our cohort (Supplementary Table 4, Fig. 2a and Extended Data Fig. 3a–g). Our inflammation score (derived from histologically inflamed samples) highly correlated with a recently described molecular inflammation score (*R* = 0.89, *P* < 2.2 × 10^−16^) (Extended Data Fig. 3h)^30^. Our score was comparable between inflamed CD and UC (Fig. 2b).

We identified common features in inflamed CD and UC including specific cell state expansions across the immune, fibroblast/pericyte and colonic epithelial compartments (Extended Data Fig. 3i,j and Supplementary Table 4). An IFN-responsive B cell state was more abundant in inflamed CD and UC. A similar B cell state has been described in the dextran sulfate sodium colitis mouse model and prevented mucosal healing^31^. We also observed multiple CD4^+^ *FOXP3*^+^ regulatory T cell (T_reg_) cell states enriched in inflamed CD and UC, including CD4^+^ *TWIST1*^+^ T_reg_ cells. *TWIST1* has been reported as a repressor of T effector cells^32,33^.

### Cellular correlates of endoscopy and histopathology indices

**Extended Data Fig. 4:**
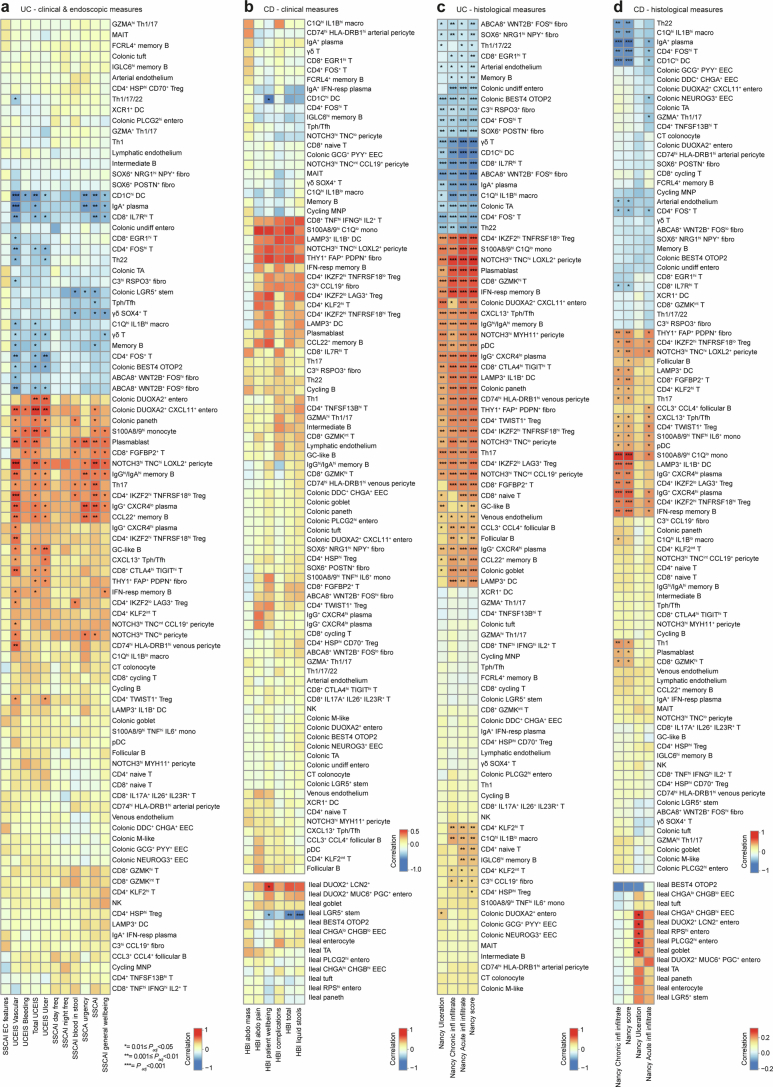
Correlation between cell state abundance, and clinical, endoscopic and histological measures of disease in CD and UC. **a-d**, Spearman correlations between cell state abundance and (**a**) SSCAI and UCEIS (**b**) HBI (**c**) Nancy score in UC and (**d**) Nancy score in CD. For (**a**), and (**b**), the maximally inflamed sample for matched endoscopic procedure used. Asterisks indicate adjusted *P* values: *=0.01 ≤ *P*_*adj*_ < 0.05, **=0.001 ≤ *P*_*adj*_ <0.01, ***=*P*_adj_<0.001. CD, Crohn’s disease; colono, colonocyte; DC, dendritic cell; EEC, enteroendocrine cell; entero, enterocyte; fibro, fibroblast; GC, germinal centre; hi, high; HBI, Harvey-Bradshaw Index; IFN-resp, interferon-responsive; ILC, innate lymphoid cell; lo, low; macro, macrophage; MAIT, mucosal-associated invariant T; MNP, mononuclear phagocyte; mono, monocyte; NK, natural killer cells; pDC, plasmacytoid dendritic cell; RPS^hi^, ribosomal protein S-high; SSCAI, simple clinical colitis activity index; TA, transit-amplifying; Tfh, CD4^+^ follicular helper T cell; Tph, CD4^+^ peripheral helper T cell; Th, CD4^+^ T helper cell; Treg, CD4^+^ regulatory T cell; UCEIS, ulcerative colitis endoscopic index of severity; UC, ulcerative colitis; Undiff, undifferentiated.

To establish the clinical relevance of scRNA-seq, we investigated correlations between cell state abundance and clinical and endoscopic disease measures. Greater concordance between the Simple Clinical Colitis Activity Index^34^ (SSCAI, UC) and cell state abundance was observed than for the Harvey-Bradshaw Index^35^ (HBI, CD), and we found 26 cell states correlated with endoscopic disease activity, the Ulcerative Colitis Endoscopic Index of Severity^36^ (UCEIS) (Extended Data Fig. 4a,b and Supplementary Table 4). We leveraged paired scRNA-seq haematoxylin-eosin (H&E) images (*n* = 204 samples) to identify over 30 cellular correlates of the histopathological Nancy index^37^ (Extended Data Fig. 4c,d). Overall, cell state abundances showed more correlations with histological inflammation features compared to clinical or endoscopic outcome measures.

### CD and UC differ by lymphocytic and epithelial stoichiometry

Given distinct clinical and histopathological features in CD and UC, we investigated differences between them (Fig. 2c–f). In inflamed CD, we observed a specific expansion of Th1 cells (Fig. 2c). Differential cell-cell interaction analyses revealed CD-specific Th-derived *IFNG* signalling to macrophages (Fig. 2g). Epithelial remodelling in CD consisted of enrichment of *PLCG2*^hi^ enterocytes (Fig. 2f). Missense variants of *PLCG2*, which encodes a phospholipase enzyme, are associated with IBD^38^ and result in intestinal inflammation^39,40^. Although associated with B cell development and tuft cells in health^41^, our findings indicate a specific relevance of *PLCG2* to enterocytes in CD. Analyses comparing the inflamed ileum and colon in CD revealed that most DEGs in the ileum were in the myeloid, stromal and epithelial compartments (Supplementary Table 4). IgG^+^ plasma cell expansion was seen in inflamed CD and UC but more pronounced in the latter (Fig. 2e). Similarly, Th17 cells were more abundant in inflammation in both diseases but more pronounced in UC (Fig. 2c). A CD8^+^ *CTLA4*^hi^ *TIGIT*^hi^ T cell state was specifically increased in inflamed UC (Fig. 2d).

**Extended Data Fig. 5:**
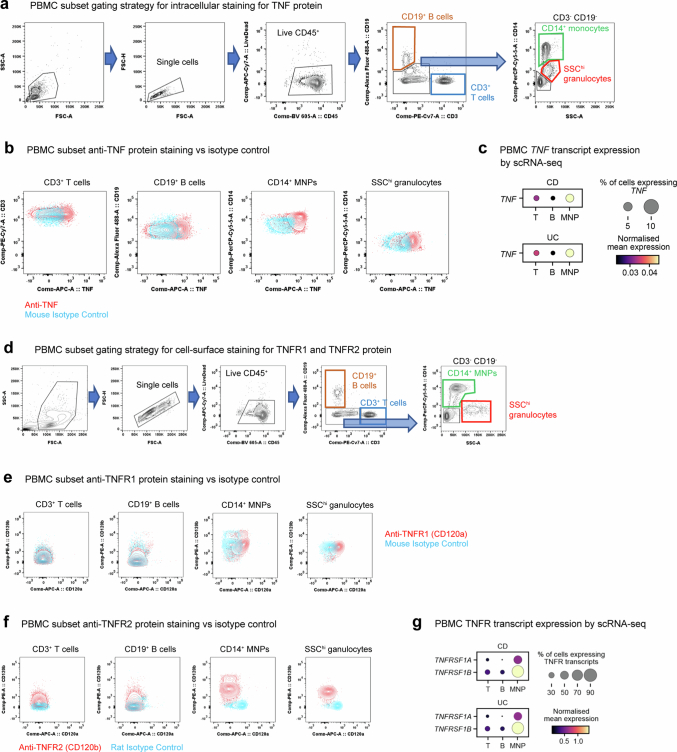
Expression of TNF and its receptors at the RNA and protein level in PBMCs. **a**, PBMC subset gating strategy for intracellular staining of TNF protein. **b**, PBMC subset anti-TNF staining. **c**, *TNF* expression by scRNA-seq in PBMCs from patients with CD (top) and UC (bottom). **d**, PBMC subset gating strategy for cell surface staining of TNFR1 and TNFR2. **e**, PBMC subset anti-TNFR1 staining. **f**, PBMC subset anti-TNFR2 staining. **g**, *TNFRSF1A* and *TNFRSF1B* expression by scRNA-seq in PBMCs from patients with CD (top) and UC (bottom). MNP, mononuclear phagocyte; SSC, side scatter.

Given the use of adalimumab in CD and UC, we next characterised the expression of *TNF* and its receptors (*TNFRSF1A* and *TNFRSF1B*, encoding TNFR1 and TNFR2, respectively). During inflammation, mean *TNF* expression per cell was highest in monocytes and CD4^+^ memory T cells (Fig. 2h). However, as the latter cells are approximately five times more abundant than monocytes, they are the top *TNF* source. Although thought to be ubiquitously expressed^42^, *TNFRSF1A* was mainly found in epithelial, stromal and myeloid cells. *TNFRSF1B* was preferentially expressed in immune cells. We confirmed this spatially using RNAScope: *TNFRSF1A* had an epithelial and lamina propria distribution, whereas *TNFRSF1B* localised to the latter (Fig. 2i). As myeloid cells had the highest expression of both receptors amongst immune cells, we assessed if this was also observable in the blood. scRNA-seq analysis of 95,134 mononuclear cells from 14 biologic-naive IBD patients revealed an analogous pattern that was also confirmed at the protein level (Extended Data Fig. 5). We also quantified TNF signalling by PROGENy analysis^43,44^. TNF signalling pretreatment in inflamed gut samples was higher in CD4^+^ T helper, myeloid, stromal and selected epithelial cells (Fig. 2h).

Collectively, this cellular census revealed substantial similarities across CD and UC, including TNF pathway gene distribution, but also key differences in lymphoid and epithelial cells.

### Inflammatory hubs map to distinct CD and UC spatial niches

**Fig. 3:**
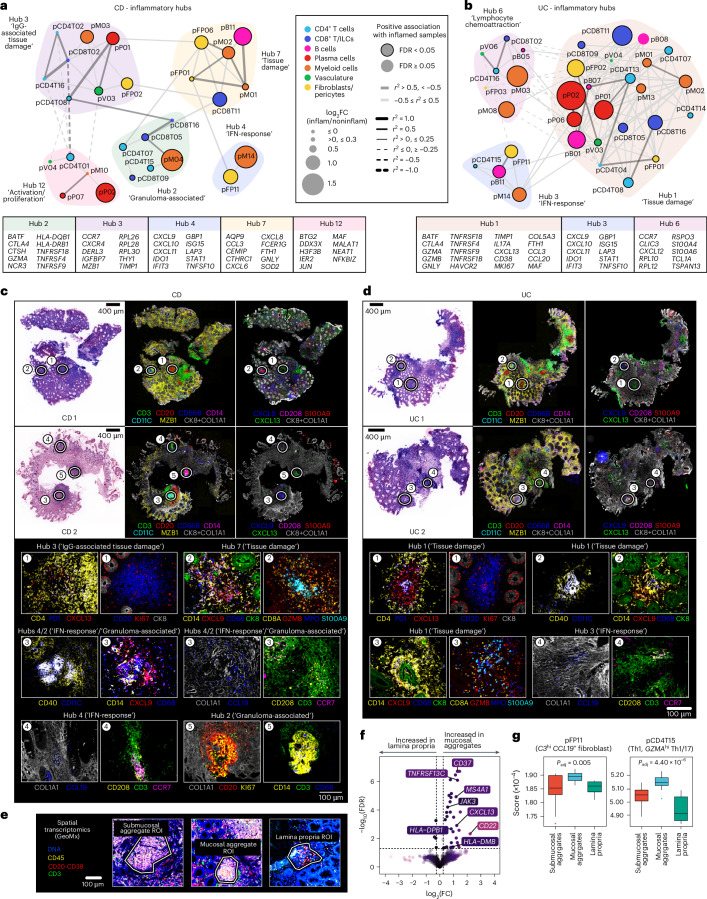
Hubs of gene expression programmes are associated with spatial niches in CD and UC. **a,b**, Network graph of covarying GEPs that constitute inflammatory hubs in (**a**) CD and (**b**) UC. Common weighted genes (within top 50) across constituent GEPs within hubs are shown below network graph. See Supplementary Table 5 for full list of cNMF GEPs in IBD and associated GO term enrichment in GEPs. **c,d**, Virtual H&E with multiplexed imaging highlighting representative regions of tissue and associated protein markers in (**c**) CD (*n* = 4 patients) and (**d**) UC (*n* = 5 patients). Sections shown from two patients from each disease. **e**, Representative GeoMx image of ROIs across submucosal aggregates (15 ROIs), mucosal aggregates (16 ROIs) and lamina propria (10 ROIs) from IBD samples, with antibody staining for CD45, CD3, CD20-CD38. **f**, Differential gene expression comparing mucosal aggregates (16 ROIs) and lamina propria (10 ROIs). **g**, GEP projection onto GeoMx samples; ROI numbers as in **f**, submucosal aggregates (15 ROIs). Boxplots show median, first (lower hinge) and third (upper hinge) quartiles; whiskers show 1.5× interquartile range. Kruskal-Wallis one-way analysis of variance conducted with subsequent pairwise testing with Wilcoxon rank-sum. DC, dendritic cell; FC, fold change; IL, innate lymphoid; pB, B cell GEP; pCD4T, CD4^+^ T cell GEP; pCD8T, CD8^+^ T cell/NK GEP; pFP, fibroblast and pericyte GEP; pM, myeloid cell GEP; pP, plasma cell GEP; pV, vascular cell GEP; ROI, region of interest.

**Extended Data Fig. 6:**
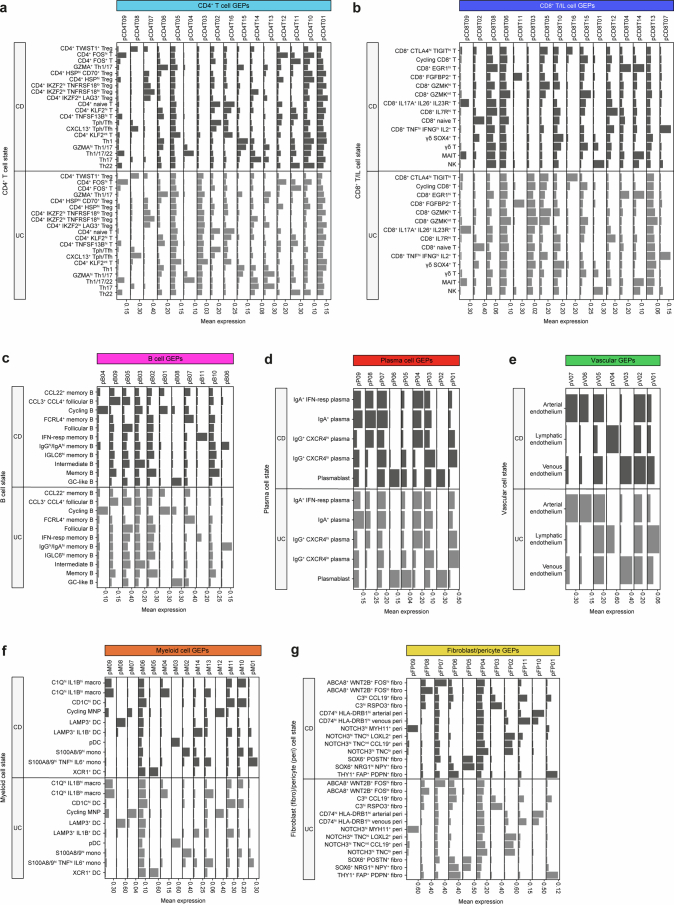
Enrichment of GEPs across cell states in the gut. cNMF was used to derive GEP score for individual cells from inflamed samples with CD and UC in (**a**) CD4^+^ T, (**b**) CD8^+^ T, (**c**) B, (**d**) plasma, (**e**) vascular, (**f**) myeloid, and (**g**) fibroblast and pericyte cells. Mean expression of GEP quantified per cell state is plotted. pB: B cell GEP; pCD4T: CD4^+^ T cell GEP; pCD8T: CD8^+^ T cell/NK GEP; pFP: fibroblast and pericyte GEP; pM: myeloid cell GEP; pP: plasma cell GEP; pV: vascular cell GEP. DC, dendritic cell; fibro, fibroblast; GC, germinal centre; hi, high; IFN-resp, interferon-responsive; ILC, innate lymphoid cell; lo, low; macro, macrophage; MAIT, mucosal-associated invariant T; MNP, mononuclear phagocyte; mono, monocyte; NK, natural killer cell; pDC, plasmacytoid dendritic cell; peri, pericyte; Tfh, CD4^+^ follicular helper T cell; Tph, CD4^+^ peripheral helper T cell; Th, CD4^+^ T helper cell; Treg, CD4^+^ regulatory T cell.

**Extended Data Fig. 7:**
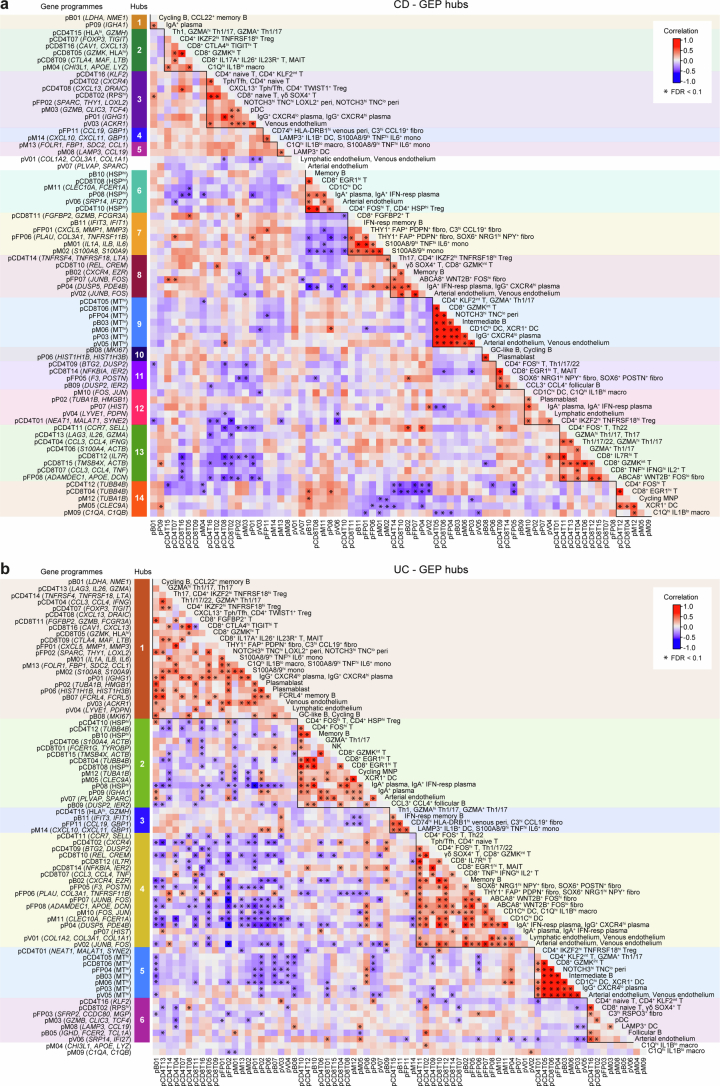
Covarying GEPs in inflamed samples with CD and UC. **a,b**, Correlogram demonstrating significant correlations (asterisks denote FDR < 0.1) between GEPs across cell compartments in inflamed samples with CD (**a**) and UC (**b**). Lines demarcate hubs. A module detection algorithm used for signed graphs was leveraged to detect hubs from a graph consisting of significantly correlated GEPs (nodes) and associated fisher-transformed correlations (edges). DC, dendritic cell; fibro, fibroblast; GC, germinal centre; hi, high; HSP, heat-shock proteins; IFN-resp, interferon-responsive; ILC, innate lymphoid cell; lo, low; macro, macrophage; MAIT, mucosal-associated invariant T; MNP, mononuclear phagocyte; mono, monocyte; MT^hi^, mitochondrial-high; NK, natural killer cell; pB, B cell GEP; pCD4T, CD4^+^ T cell GEP; pCD8T, CD8^+^ T cell/NK GEP; pDC, plasmacytoid dendritic cell; peri, pericyte; pFP, fibroblast and pericyte GEP; pM, myeloid cell GEP; pP, plasma cell GEP; pV, vascular cell GEP; RPS^hi^, ribosomal protein S-high; Tfh, CD4^+^ follicular helper T cell; Tph, CD4^+^ peripheral helper T cell; Th, CD4^+^ T helper cell; Treg, CD4^+^ regulatory T cell.

As partitioning cells into discrete cell states may not capture the full spectrum of cell identity and activity, we leveraged consensus non-negative matrix factorisation (cNMF) to identify gene expression programmes (GEPs) within cell types^45^. GEPs can represent cell identity but can also reflect activation processes concurrently occurring within a cell (Supplementary Table 5, Supplementary Fig. 1 and Extended Data Fig. 6). We assessed each cell compartment to identify inflammation-associated GEPs and examined correlations between GEPs. Groups of highly correlated GEPs, termed hubs, may represent participants in related biological processes. We derived 14 hubs in CD and 6 in UC (Extended Data Fig. 7). Hubs in which more than 50% of GEPs were enriched in inflammation were deemed ‘inflammatory’ (Fig. 3a,b).

In both CD and UC, we observed two IFN-response hubs: hub 4 and hub 3, respectively (Fig. 3a,b). These were enriched for type I and II IFN-response pathways (Supplementary Table 5). Within these hubs, myeloid (pM14) and fibroblast/pericyte (pFP11) GEPs were shared between CD and UC (Fig. 3a,b). pM14 was enriched in *LAMP3*^+^ *IL1B*^+^ DCs and to a lesser extent, S100A8/9^hi^ *TNF*^hi^ *IL6*^+^ monocytes (Extended Data Fig. 6). pFP11 included the follicular reticular marker *CCL19*, trafficking molecules (*MADCAM1*), selectins *(SELE*) and MHC class II. Enrichment of this GEP was observed in *C3*^hi^ *CCL19*^+^ fibroblasts and *CD74*^hi^ *HLA-DRB1*^hi^ venous pericytes in both diseases (Extended Data Fig. 6).

We used the CCL19 (pFP11) and CXCL9 (pM14 and pFP11) protein markers to localise the shared GEPs spatially within matched biopsy sections. CCL19 was expressed on COL1A1^+^ stromal cells (pFP11) and on LAMP3^+^ CCR7^+^ DCs present in CD3^+^ T cell aggregates (Fig. 3c,d, region 4). This DC was described by pM08 (*LAMP3*, *CCR7*, *CCL19*) (Extended Data Figs. 6, 7) and enriched in inflamed CD and UC (Supplementary Table 5). CXCL9 was also found in T cell aggregates, expressed on CD14^+^ CD40^hi^ CD11c^+^ monocyte-derived DCs (Fig. 3c, region 3). These DCs were additionally situated around damaged epithelial crypt cells (Fig. 3d, region 2). The CXCL9 expression pattern suggests IFN signalling is associated with inflammation and can be found in T cell aggregates and/or regions of epithelial damage in both diseases.

Shared GEPs were also seen in hub 7 (CD) and hub 1 (UC), including pCD8T11, pM02 and pFP01. These GEPs mapped to CD8^+^ *FGFBP2*^+^ T cells, monocytes and *THY1*^*+*^ *FAP*^*+*^ *PDPN*^*+*^ activated fibroblasts, respectively. *GZMB*, encoding granzyme B, is a marker of CD8^+^ *FGFBP2*^+^ T cells (Extended Data Fig. 1d). The GZMB^+^ CD8A^+^ T cells localised to areas of epithelial (CK8^+^) damage (Fig. 3c, region 2, and Fig. 3d, region 3), proximal to S100A9^+^ MPO^+^ CD66B^+^ neutrophil aggregates and CXCL9^+^ monocyte-derived DCs. This suggests that in epithelial damage, CD8^+^ *FGFBP2*^+^ T cells, potently expressing *IFNG* (Extended Data Fig. 1d), may drive the monocyte-derived DC IFN-response. We previously described a neutrophil-stromal interaction in epithelial damage regions^28^. Here, we extended our observations by also localising a GZMB^+^ CD8^+^ T cell state to these regions.

CD hub 2 also shared multiple GEPs with UC hub 1: pCD4T07, pCD8T16, pCD8T05 and pCD8T09. Notably, two GEPs (pM04, pCD4T15) present in CD hub 2 were absent in UC hub 1. pM04 was most expressed in resident C1Q^hi^ *IL1B*^lo^ macrophages (Extended Data Fig. 6f). pM04 top genes included *CHI3L1*, *CYP27A1, APOE* and *CTSD* (Supplementary Fig. 1). GO term enrichment highlighted terms relating to cholesterol homeostasis and lysosomal transport (Supplementary Table 5). This gene signature was recently described in granulomatous macrophages in sarcoidosis-affected skin^46^. pCD4T15 mapped to Th1 and Th1/17 cells which have also been implicated in sarcoidosis granulomas (Supplementary Table 5 and Extended Data Fig. 6a)^46^. This suggests hub 2 as representative of granulomas, seen specifically in CD (Fig. 3c, regions 3 and 5).

In UC, which is not a granulomatous condition, pCD4T15 was instead strongly correlated with pFP11 within the IFN-response hub 3. Using the GeoMx spatial platform we assessed the transcriptomic differences between the lamina propria and lymphoid aggregates (Fig. 3e and Supplementary Table 5). We identified higher expression of MHC Class II genes alongside *CXCL13* in mucosal aggregates (Fig. 3f). Higher expression of *TNFRSF13C* and *MS4A1* were indicative of a pro-B cell environment. In addition, mucosal aggregates were enriched for pFP11 and pCD4T15 (Fig. 3g).

### Epithelial and myeloid features predicate anti-TNF outcome

**Fig. 4:**
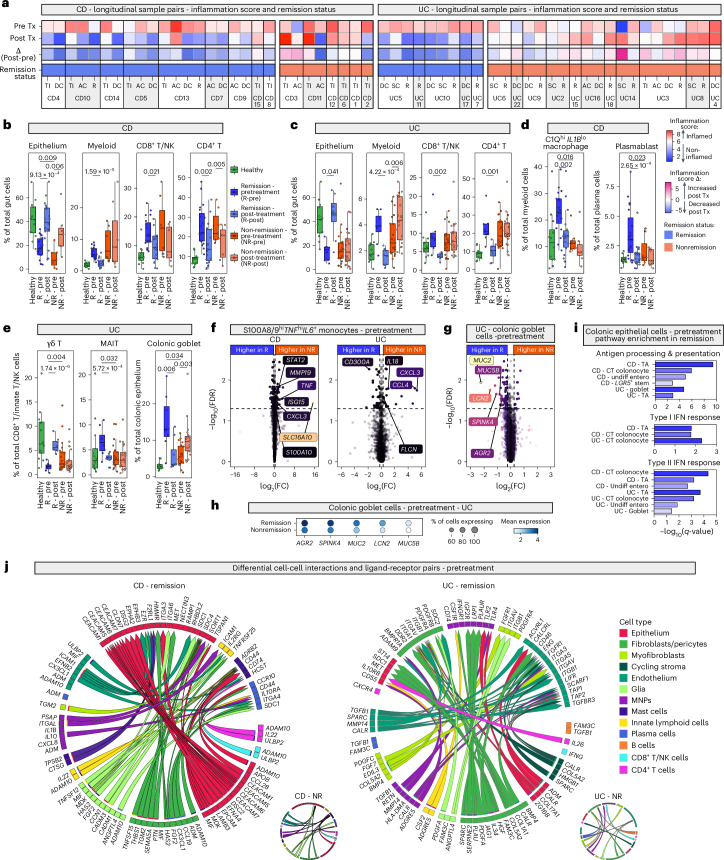
Pretreatment differences in remission and nonremission patient groups in CD and UC. **a**, Heatmap showing inflammation score for paired pre- and post-treatment samples (CD and UC). R, rectum; Tx, treatment. **b**, Boxplots showing proportion of cell compartment out of total cells in samples from 3 healthy individuals (12 samples), 10 CD remission patients (19 pretreatment and 19 post-treatment samples) and 5 CD nonremission patients (7 pretreatment and 7 post-treatment samples). Boxplots show median, first (lower hinge) and third (upper hinge) quartiles; whiskers show 1.5× interquartile range (**b**–**e**). Differential abundance testing at baseline and longitudinally using MASC (**b**–**e**). For baseline, only inflamed samples were included. BH-adjusted *P* values (two-sided) shown. **c**, Boxplots showing proportion of cell compartment out of total cells in sample across 3 healthy individuals (12 samples), 4 UC remission patients (8 pretreatment and 8 post-treatment samples) and 13 UC nonremission patients (21 pretreatment and 21 post-treatment samples). **d**, Boxplots showing proportion of cell state out of total myeloid cells (left) and total plasma cells (right). Sample numbers as in (**b**). **e**, Boxplots showing proportion of cell state out of total CD8^+^ T/innate T/NK cells (left and middle) and total colonic epithelium (right). Sample numbers as in (**c**). **f**, Differential expression comparing pretreatment remission and nonremission S100A8/9^hi^ *TNF*^hi^ *IL6*^+^ monocytes using MAST. Sample numbers as in (**b**) and (**c**) for CD and UC, respectively. **g**, Differential expression comparing pretreatment colonic goblet cells between remission and nonremission in UC using MAST. Sample numbers as in (**c**). **h**, Dotplot showing select genes in pretreatment colonic goblet cells in UC remission and nonremission subgroups at baseline using MAST. Sample numbers as in (**c**). **i**, Gene set enrichment analysis conducted on differential expression analysis of pretreatment samples in CD and UC comparing remission and nonremission epithelial cell states. **j**, Cell-cell interaction plots showing ligand-receptor pairs enriched in remission at baseline in CD (left) and UC (right). Insets show respective nonremission plots. AC, ascending colon; CT, crypt top; DC, descending colon; FC, fold change; IFN, interferon; MAIT, mucosal-associated invariant T cells; MNP, mononuclear phagocytes; NK, natural killer cells; NR, nonremission; SC, sigmoid colon; R, remission; TA, transit-amplifying cells; TI, terminal ileum; Tx, treatment; Undiff entero, undifferentiated enterocyte.

We next characterised differences at baseline in patients achieving remission and those who did not, after adalimumab. At baseline, inflammation score was not associated with future remission status in our cohort (all *P* ≥ 0.05) (Fig. 4a).

In CD, baseline epithelial cell frequency was significantly higher in remission compared to nonremission groups. Epithelial cells increased following adalimumab, irrespective of remission status (Fig. 4b). However, only in remission was the post-treatment frequency analogous to healthy samples. This difference was not observed in other cell types or in UC (Fig. 4b,c and Supplementary Table 6). We then investigated differences at the cell-state resolution.

In the myeloid compartment, we found an increased baseline abundance of C1Q^hi^ *IL1B*^lo^ macrophages associated with CD remission (Fig. 4d). A key marker for these cells, *TREM2*, is associated with pro-repair/remission in RA (Supplementary Table 2)^18^. pM04, specific to these cells, was enriched for genes relating to negative regulation of TNF production (*ACP5*, *LILRB4*, *GPNMB*, *TREM2*, *TSPO*) (Supplementary Table 5). Consistent with a pro-remission role, these cells had low abundance in health and at baseline in the nonremission group. A similar abundance pattern was observed for plasmablasts (CD) and MAIT cells (UC) (Fig. 4d,e and Supplementary Table 6). In UC, colonic goblet cells were most abundant in the remission group at baseline but increased following treatment in nonremission (Fig. 4e). Analogous results were seen in colonic CD. Conversely, $\gamma \delta$ T cells were significantly lower in abundance at baseline in the UC remission group (Fig. 4e).

We then conducted differential expression analysis at baseline in the remission/nonremission groups (Supplementary Table 7). Notably in CD and UC, we found baseline differences in gene expression within S100A8/9^hi^ *TNF*^hi^ *IL6*^+^ monocytes (Fig. 4f). In CD nonremission, these cells had higher expression of chemokines (*CXCL3*) and cytokines (*TNF*), and exhibited IFN-response. In UC, similar DEGs (*CXCL3*, *IL18*) were observed in the nonremission group, whereas expression of inhibitory receptor, *CD300A*, was higher in remission. GEPs enriched in these cells in CD (pM01) and UC (pM01, pM13) were found in hub 7 and 1, respectively, both in tissue damage areas (Supplementary Fig. 1 and Fig. 3a,b).

In UC, we observed baseline DEGs that distinguished goblet cells in remission/nonremission (Fig. 4g). Mucin (*MUC2*, *MUC5B*) expression was higher in remission (Fig. 4g,h). Interestingly, in both CD and UC, MHC class I and II and IFN-response genes were enriched in remission across multiple epithelial cell states (Fig. 4i and Supplementary Table 7). Differential cell-cell interaction also revealed a prominent role for epithelial-epithelial, epithelial-stromal and myeloid interactions at baseline in the remission groups (Fig. 4j).

### Specific cellular profiles underpin anti-TNF nonremission

Following adalimumab treatment, remission was characterised by epithelial reconstitution and a concomitant immune cell decrease (Fig. 4b,c). In nonremission, epithelial increases were insufficient or nonexistent, and immune cells showed minimal changes, except for the myeloid expansion observed in UC.

**Fig. 5:**
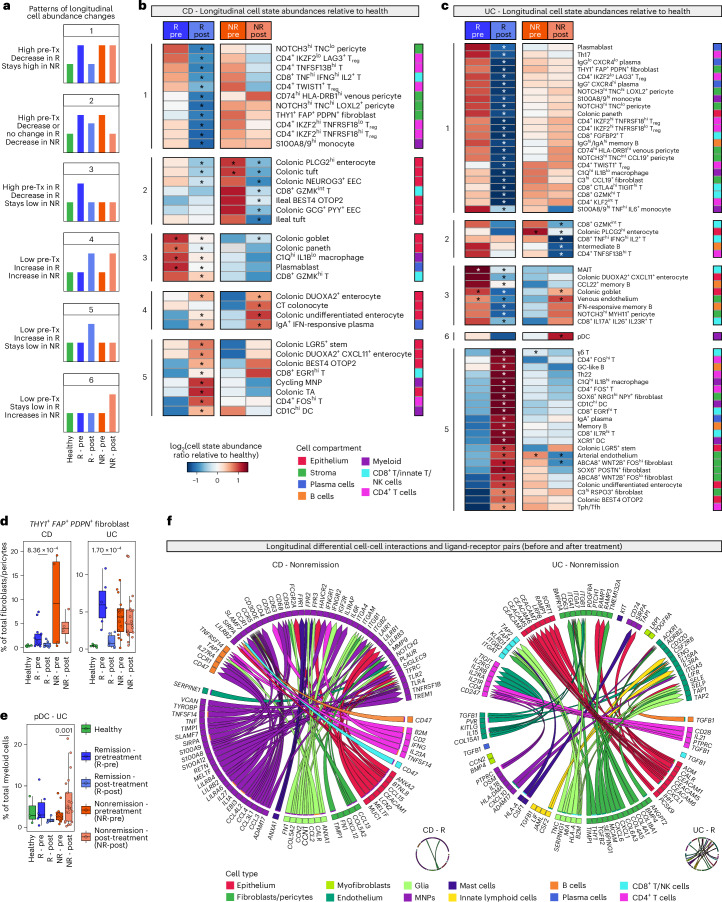
Cellular and molecular changes following adalimumab in CD and UC. **a**, Schematic showing patterns of cell abundance changes by treatment status and outcome, compared to health. **b,c**, Heatmaps showing cell state abundances by treatment status and outcome, compared to health in (**b**) CD and (**c**) UC. Pattern numbers as in (**a**). Asterisks indicate BH-adjusted *P* value < 0.05. Pretreatment, asterisks indicate significant differences at baseline between remission outcomes. Post-treatment, asterisks indicate significant differences from baseline to post-treatment. Sample numbers for **b**–**e** are as outlined in Fig. 4b,c. **d**, Boxplots showing proportion of *THY1*^+^ *FAP*^+^ *PDPN*^+^ fibroblasts out of total fibroblast/pericytes across CD (left) and UC (right) treatment and outcome groups. Boxplots show median, first (lower hinge) and third (upper hinge) quartiles; whiskers show 1.5x interquartile range (**d,e**). Differential abundance testing at baseline and longitudinally using MASC; BH-adjusted *P* values (two-sided) shown (**d,e**). **e**, Boxplots showing proportion of pDCs out of total myeloid cells across UC treatment and outcome groups. **f**, Cell-cell interaction plots showing differential ligand-receptor pairs enriched in CD (left) and UC (right) post-treatment nonremission. Insets show remission plots. DC, dendritic cell; EEC, enteroendocrine cell; GC, germinal centre; hi, high; IFN, interferon; ILC, innate lymphoid cell; int, intermediate; lo, low; MAIT, mucosal-associated invariant T; MNP, mononuclear phagocyte; mono, monocyte; NK, natural killer cells; NR, nonremission; pDC, plasmacytoid dendritic cell; R, remission; TA, transit-amplifying; Tfh, CD4^+^ follicular helper T cell; Tph, CD4^+^ peripheral helper T cell; Th, CD4^+^ T helper cell; T_reg_, CD4^+^ regulatory T cell; Tx, treatment.

Cell state abundance changes post-treatment were broadly organised into six patterns (Fig. 5a–c). Pattern 1 was characterised by cell states with high pretreatment frequency, which significantly decreased after adalimumab in remission but remained high in nonremission (for example, *THY1*^+^ *FAP*^+^ *PDPN*^+^ fibroblasts) (Fig. 5d). Pattern 2 described cells that significantly decreased after adalimumab in nonremission and were unchanged or decreased in remission (for example, CD8^+^ *GZMK*^int^ T cells). Pattern 3 had cells with high pretreatment frequency in remission that decreased after adalimumab, but were low pretreatment in nonremission. This included cells with remission-associated baseline differences (for example, colonic goblet cells, Fig. 4d,e). Pattern 4 was typified by cells in CD showing a concordant increase after adalimumab regardless of treatment outcome (for example, colonic undifferentiated enterocytes). Pattern 5 included cell states that increased post-treatment in remission but did not significantly increase in nonremission (for example, colonic *LGR5*^+^ stem cell). The final pattern was unique to plasmacytoid DCs (pDCs) in UC; a significant increase was observed post-treatment in nonremission, with no remission or baseline differences (Fig. 5e).

**Extended Data Fig. 8:**
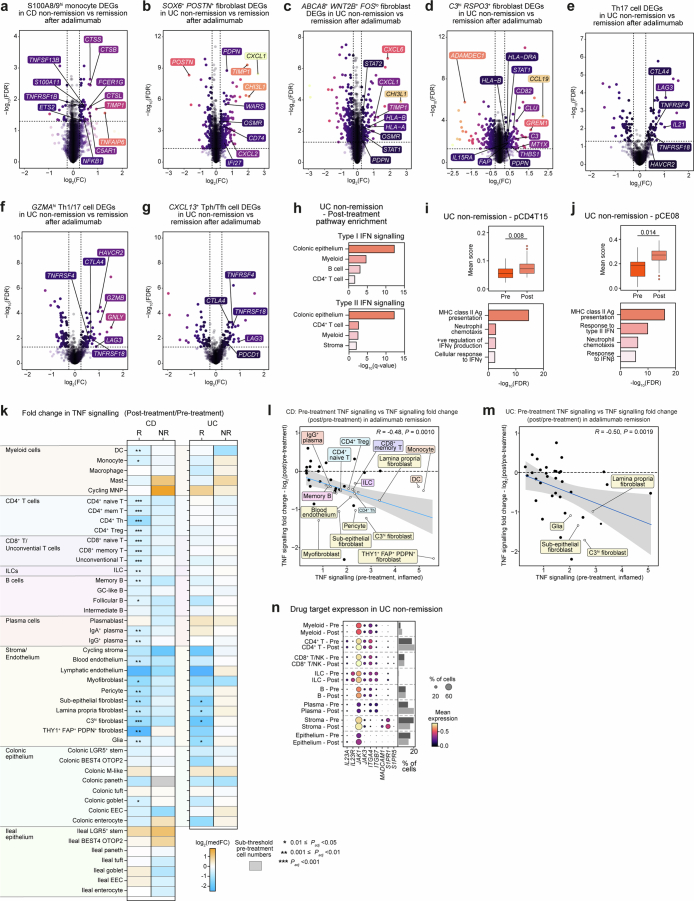
Longitudinal changes following adalimumab in CD and UC, and characterisation of the TNF pathway following adalimumab. **a-g**, Volcano plot depicting cell state-specific differentially expressed genes in (**a**) CD, and (**b-g**) UC. Negative fold-change indicates increase in remission (post-pre). Positive fold-change indicates increase in nonremission (post-pre). **h**, Gene set enrichment analysis of UC longitudinal differential expression analysis (Supplementary Table 8) across compartments. **i,j**, Boxplots showing longitudinal differences in (**i**) pCD4T15 and (**j**) pCE08 enrichment UC nonremission post-treatment (Supplementary Table 8). Sample numbers as in Fig. 4. Boxplots show median, first (lower hinge) and third (upper hinge) quartiles; whiskers show 1.5x interquartile range. Differential abundance testing longitudinally using MASC. Differential GEP enrichment tested by generalised linear mixed models. BH-adjusted *P* values (two-sided) shown. Barplots show GEP top enriched pathways (Supplementary Table 5). **k**, PROGENy was used to calculate TNF signalling scores per cell^43^. The 75^th^ percentile score for TNF signalling in each ‘intermediate’ resolution level cell type was taken as representative of individual samples. Only paired samples were used to calculate median fold change (medFC) in remission and nonremission with significance testing using lmer function as part of the lmerTest package with individual patients modelled as random effects. Asterisks indicate BH-adjusted two-sided *P* values: *=0.01 ≤ *P*_*adj*_ < 0.05, **=0.001 ≤ *P*_*adj*_ <0.01, ***=*P*_*adj*_<0.001. **l,m**, Spearman correlation between TNF signalling fold change and TNF signalling score pre-therapy in patients achieving remission after adalimumab treatment in (**l**) CD and (**m**) UC. Line indicates linear regression with 95% confidence interval (grey band), two-sided *P* values shown. **n**, Dotplot showing expression of genes associated with approved advanced therapies, before and after adalimumab in UC nonremission. Bar chart shows median abundance of compartment in context of treatment (pre/post) as a proportion of total cells in sample. Ag, antigen; DC, dendritic cell; EEC, enteroendocrine cell; FC, fold change; fibro, fibroblast; GC, germinal centre; hi, high; IFN-resp, interferon-responsive; ILC, innate lymphoid cell; int, intermediate; lo, low; MAIT, mucosal-associated invariant T; MNP, mononuclear phagocyte; mono, monocyte; NK, natural killer cells; NR, nonremission; pDC, plasmacytoid dendritic cell; R, remission; RPS^hi^, ribosomal protein S-high; TA, transit-amplifying; Tfh, CD4^+^ follicular helper T cell; Tph, CD4^+^ peripheral helper T cell; Th, CD4^+^ T helper cell; Treg, CD4^+^ regulatory T cell.

We next performed differential cell-cell interaction analysis (Fig. 5f). We observed myeloid-myeloid and CD4^+^ T cell-myeloid interactions increasing despite adalimumab in CD nonremission. In keeping with baseline nonremission-associated DEGs in S100A8/9^hi^ *TNF*^hi^ *IL6*^+^ monocytes (Fig. 4f), the nonremission cell-cell interactome was characterised by ligands including alarmins, chemokines and cytokines in the myeloid compartment. Longitudinal expression analysis utilising an interaction term for treatment and remission status demonstrated increased *TNFRSF1B*, *TREM1* and cathepsin genes in S100A8/9^hi^ monocytes post-treatment in nonremission (Extended Data Fig. 8a, Supplementary Fig. 2 and Supplementary Table 8). In C1Q^hi^ *IL1B*^hi^ macrophages, increased expression of *ETS2*, a transcription factor associated with CD and other IMIDs, was noted (Supplementary Table 8)^47^. *IFNG-IFNGR2* interactions between CD4^+^ T cells and myeloid cells were also observed in CD nonremission (Fig. 5f).

In UC nonremission, myeloid cells also exhibited increased activation with enhanced alarmins, IFN-response and cathepsin genes (Supplementary Table 8). A myeloid-vascular axis (*CXCL10*-*ACKR1*) was observed (Fig. 5f). Fibroblasts showed increased expression of ligands analogous to the activated fibroblast phenotype (*THY1, CXCL1*, *CXCL6*). Longitudinal expression analysis identified *THY1*, *PDPN*, *OSMR* and potent neutrophil chemoattractants (*CXCL1*, *CXCL6*) as increased in fibroblasts localising to the sub-epithelial region (*SOX6*^+^ *POSTN*^+^ fibroblasts) and lamina propria (*ABCA8*^+^ *WNT2B*^+^ *FOS*^hi^ and *ABCA8*^+^ *WNT2B*^+^ *FOS*^lo^ fibroblasts), after adalimumab treatment in nonremission (Extended Data Fig. 8b,c and Supplementary Table 8). Expansion of THY1^+^ FAP^+^ synovial fibroblasts has been previously associated with RA, suggesting that this is a cross-IMID pathogenic fibroblast^16,48^. In fibroblasts near the intestinal stem cell niche^14^ (*C3*^hi^ *RSPO3*^+^ fibroblasts), we saw upregulation of the T cell attractant *CCL19* in UC nonremission (Extended Data Fig. 8d).

In the CD4^+^ T cell compartment, following adalimumab treatment, we found increased signalling including *IL21*-*IL21R* interactions in UC nonremission (Fig. 5f). Upregulated *IL21, TNFRSF1B* and immune checkpoint genes (*LAG3*, *CTLA4*, *TNFRSF4*, *TNFRSF18*, *HAVCR2*) were seen in Th17 cells (Extended Data Fig. 8e). These checkpoint genes and cytotoxic genes were expressed in *GZMA*^hi^ Th1/17 cells (Extended Data Fig. 8f). *PDCD1* and other checkpoint genes were also upregulated in *CXCL13*^+^ T peripheral helper (Tph)/T follicular helper (Tfh) cells (Extended Data Fig. 8g).

Multicompartmental IFN-response was seen in UC nonremission (Extended Data Fig. 8h and Supplementary Table 8). Two IFN-associated GEPs (pCD4T15 and a colonic epithelial GEP, pCE08) were increased in this patient group (Extended Data Fig. 8i,j and Supplementary Table 8). Notably, pDCs, the main producers of type I IFN, were specifically expanded post-treatment in nonremission (Fig. 5e and Supplementary Table 6).

Using PROGENy, we observed a significant reduction in TNF signalling in remission in CD (immune cells and stroma) and UC (stroma only). In CD and UC remission, reductions were specifically seen in *THY1*^+^ *PDPN*^+^ *FAP*^+^ fibroblasts. Cells with the greatest post-treatment decrease in TNF signalling had high signalling levels at baseline (Extended Data Fig. 8k–m).

These findings suggest that nonremission after adalimumab is strongly associated with worsening of disease at the cellular level. This indicates a need to promptly switch to alternative therapies in nonresponding patients, guided by the post-treatment cellular/molecular landscape (Extended Data Fig. 8n and Supplementary Figs. 3 and 4).

### Shared IMID pathways associate with RA lymphoid pathotype

**Fig. 6:**
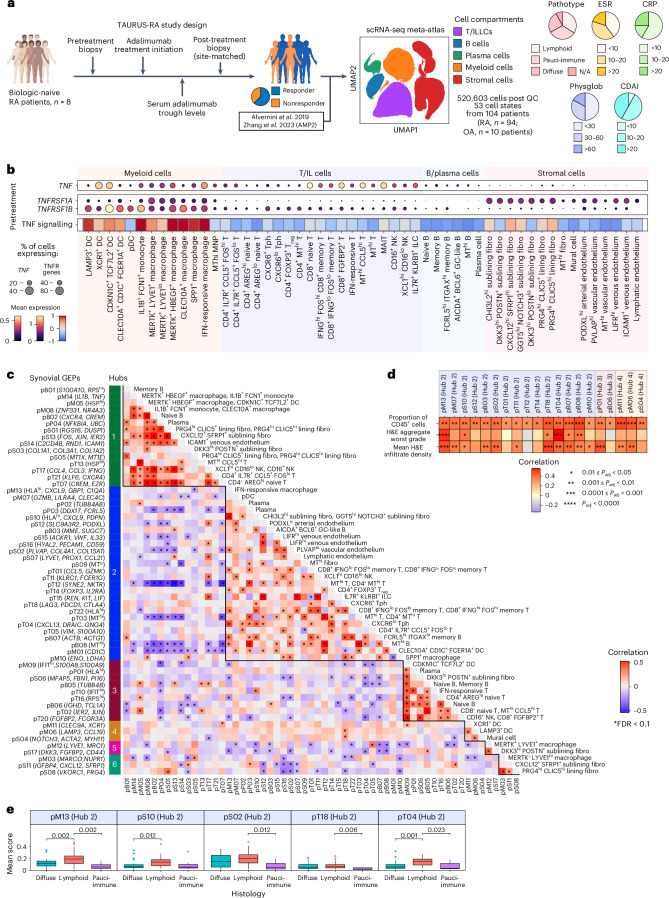
Inflammatory pathways shared between IBD and RA are associated with the lymphoid pathotype in the joint. **a**, TAURUS-RA study design and integration with external datasets to create a synovial tissue meta-atlas^18,20^. **b**, Mean mRNA transcript expression at the cell-state resolution is shown for *TNF*, *TNFRSF1A* and *TNFRSF1B* in inflamed RA samples. TNF signalling scores in inflamed RA samples by PROGENy^43^. Heatmap shows relative enrichment of TNF signalling scores. **c**, Gene expression programme (GEP) correlations. Asterisk indicates significantly correlated GEP pairs (*P*_*adj*_ < 0.1). Solid lines demarcate highly correlated GEP hubs. **d**, Only AMP2 samples included in this analysis as only this dataset had H&E aggregate grading and infiltrate density. Spearman correlations between GEP expression and proportion of CD45^+^ cells per sample, worst grade of aggregates and mean infiltration as indicated by associated H&E with BH correction for GEP numbers within cell compartments. Number of asterisks indicates significance level (two-sided): *0.01 ≤ *P*_*adj*_ < 0.05, **0.001 ≤ *P*_*adj*_ < 0.01, ***0.0001 ≤ *P*_*adj*_ < 0.001, ****P_*adj*_ < 0.0001. **e**, Associations between GEP expression and histological pathotypes. Only AMP2 data were included in this analysis; diffuse (*n* = 30 patients), lymphoid (*n* = 33 patients) and pauci-immune (*n* = 7 patients) pathotypes. Boxplots show median, first (lower hinge) and third (upper hinge) quartiles; whiskers show 1.5x interquartile range. Kruskal-Wallis one-way analysis of variance conducted to test association between GEPs within cell compartments which were positively correlated with proportion of CD45^+^ cells, with FDR correction for GEP number within cell compartments. Pairwise Wilcoxon rank-sum tests only conducted for significant GEPs, with FDR correction for pairwise comparisons between histological pathotypes. Significant adjusted *P* values displayed above relevant comparisons. CRP, C-reactive protein; CDAI, clinical disease activity index; DC, dendritic cell; ESR, erythrocyte sedimentation rate; fibro, fibroblast; GC, germinal centre; HSP^hi^, heat shock protein-high; IFIT^hi^, Interferon induced proteins with tetratricopeptide repeat genes-high; ILC, innate lymphoid cell; MAIT, mucosal-associated invariant T; MNP, mononuclear phagocyte; MT^hi^, mitochondrial-high; NK, natural killer; OA, osteoarthritis; pB, B cell GEP; pDC, plasmacytoid dendritic cell; physglob, physician global assessment RA; pM, myeloid cell GEP; pP, plasma cell GEP; pS, stromal cell GEP; pT, T/NK cell GEP; RPS^hi^, ribosomal protein S-high; Tph, CD4^+^ peripheral helper T cell; T_reg_, CD4^+^ regulatory T cell.

**Extended Data Fig. 9:**
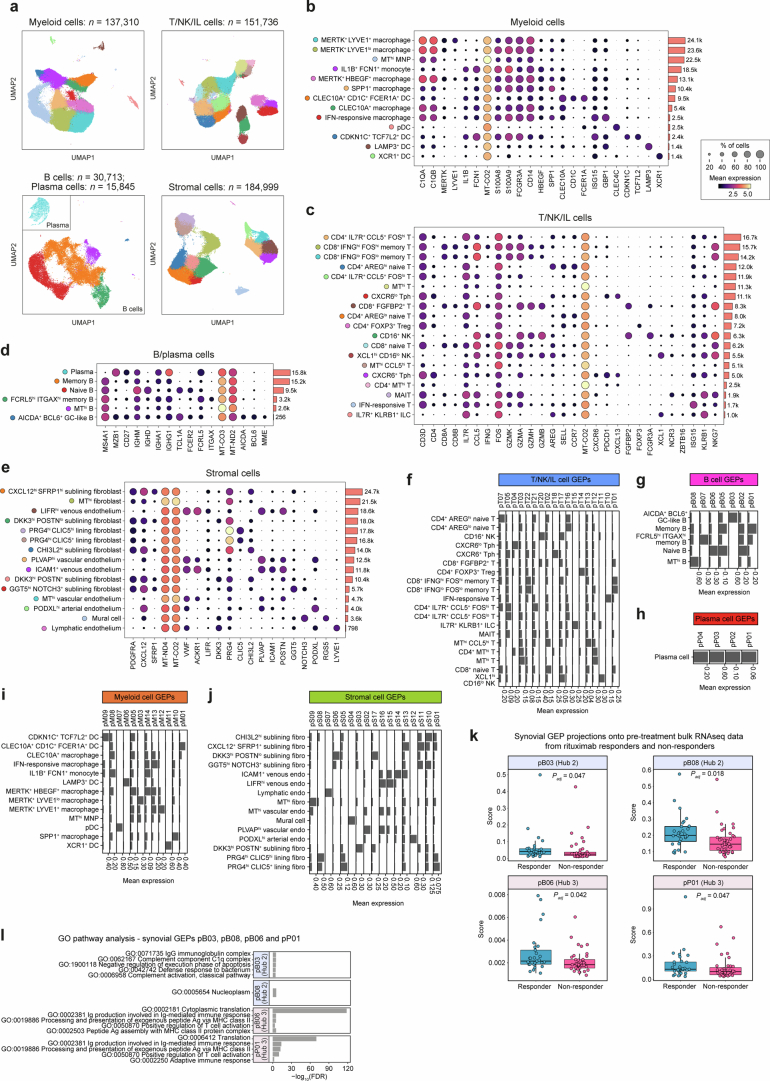
Synovial cell states, GEP enrichment of GEPs across cell states and treatment response associations. **a**, Uniform manifold approximation and projections (UMAPs) of cell states in the scRNA-seq dataset. **b-e**, Dotplots showing the expression of cell state marker genes in (**b**) myeloid cells, (**c**) T/NK/IL cells, (**d**) B/plasma cells, and (**e**) stromal cells. **f**-**j**, cNMF was used to derive GEP scores for individual cells from inflamed samples with RA in (**f**) T/NK/IL, (**g**) B, (**h**) plasma, (**i**) myeloid, and (**j**) stromal cells. Mean expression of GEP quantified per cell state. **k**, Baseline visit samples in the R4RA study were selected for analysis; GEPs positively correlated with inflammation were tested for association with therapy nonresponse. Boxplots show median, first (lower hinge) and third (upper hinge) quartiles; whiskers show 1.5x interquartile range. Wilcoxon signed-rank test used to test for significance (*P*_*adj*_ < 0.05, two-sided) between responders (29 patients) and non-responders (39 patients) to rituximab at baseline (also see Supplementary Table 9). **l**, GO term enrichment for GEPs associated with clinical response to rituximab. GO terms were generated by GOATOOLS overrepresentation analysis of the top 150 weighted genes in constituent GEPs. All genes tested were used as the gene universe. See Supplementary Table 9 for full list of cNMF GEPs in RA and associated GO term enrichment in GEPs. DC, dendritic cell; fibro, fibroblast; GC, germinal centre; hi, high; IFN-resp, interferon-responsive; ILC, innate lymphoid cell; lo, low; macro, macrophage; MAIT, mucosal-associated invariant T; MNP, mononuclear phagocyte; mono, monocyte; MT^hi^, Mitochondrial-high; NK, natural killer cell; pDC, plasmacytoid dendritic cell; Tfh, CD4^+^ follicular helper T cell; Tph, CD4^+^ peripheral helper T cell; Treg, CD4^+^ regulatory T cell.

Shared efficacy to anti-TNF across IMIDs suggests shared pathological mechanisms. Therefore, we determined whether the cellular hubs and interactions identified in IBD might underpin inflammation and hold implications for drug response in RA. We recruited patients before and after adalimumab treatment (*n* = 8 patients with paired samples from *n* = 4) (Fig. 6a, Supplementary Table 1). Whole digestion of synovial tissue followed by scRNA-seq yielded 65,588 high-quality single-cell transcriptomes. Integrating our data with other whole-digested synovial datasets^18,20^ resulted in a 520,603-cell meta-atlas (Fig. 6a and Extended Data Fig. 9a–e).

*TNF* expression was highest in myeloid and T cells (Fig. 6b). Like IBD, prominent *TNFRSF1A* expression was seen in stromal cells, whereas *TNFRSF1B* expression was highest in immune cells. Consistent with our gut findings, TNF signalling was highest in myeloid cells and fibroblasts in the RA synovium.

Next, we derived cNMF profiles within each cell compartment and associated hubs for RA (Fig. 6c, Extended Data Fig. 9f–j, Supplementary Fig. 5 and Supplementary Table 9). Twenty out of 58 GEPs across six hubs positively correlated with inflammation, using a recently developed inflammation score^49^ (Fig. 6d,e and Supplementary Table 9). Fourteen GEPs correlated with infiltrate density. Of these, five were associated with aggregates (worst grade) (Fig. 6d). All GEPs enriched in lymphoid pathotype patients were found in hub 2 (Fig. 6e).

Like hubs 4 (CD) and 3 (UC) in IBD, genes in multiple GEPs across cell compartments in RA hub 2 (pM13, pS10, pT22), specifically indicated IFN-response, and B cell activation and proliferation (for example *TNFSF13B*) (Fig. 6c). pM13 was most enriched in IFN-responsive macrophages, whereas pS10 was most prominent in sublining fibroblasts (Extended Data Fig. 9i, j). Germinal centre-associated B cell (pB03) and T cell-associated GEPs facilitating B cell recruitment (pT04) and activation (pT18) were detected in *CXCR6*^lo^ and *CXCR6*^+^ Tph, respectively, suggesting that hub 2 represents a pro-B cell microenvironment.

Given the paucity of well-powered independent longitudinal cohorts examining anti-TNF response using synovial tissue, we examined GEPs in the context of rituximab therapy in RA (Supplementary Table 9)^50^. Germinal centre-associated GEP, pB03 and other B/plasma cell GEPs (pB06, pB08, pP01) were associated with rituximab response at baseline (Extended Data Fig. 9k,l).

Taken together, these findings indicate that across inflamed gut and joint, there are similarities in TNF pathway gene expression. Furthermore, lymphocyte infiltration programmes associated with IFN signalling are present across all three IMIDs we studied, suggesting that targeting IFN signalling might be considered in these diseases.

## Discussion

**Extended Data Fig. 10:**
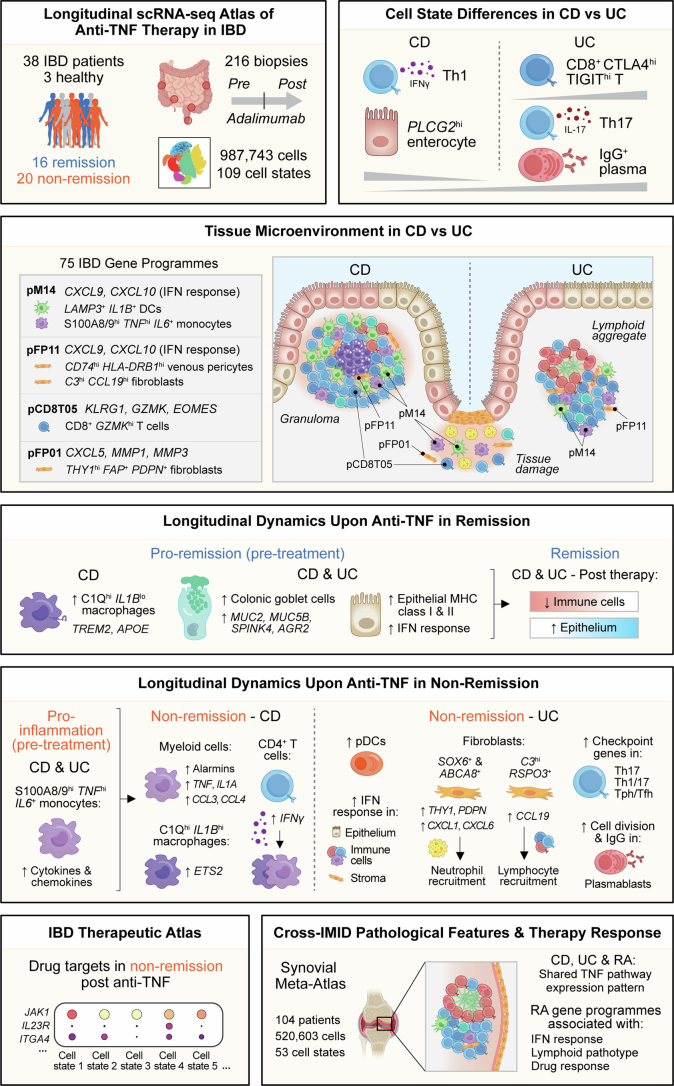
A longitudinal single-cell therapeutic atlas of adalimumab treatment in IBD. Schematic summarising the TAURUS study design and key findings. Our resource provides a longitudinal, therapeutic scRNA-seq atlas comprising ~1 million cells organised into 109 cell states from 216 gut biopsies across 41 individuals (16 remission, 20 nonremission, 3 healthy). This atlas reveals differences in gut cell state abundance that distinguish CD and UC. Using a systems-biology approach we identify hubs of multi-cellular communities, based on 75 IBD gene programmes, which localise to distinct tissue microenvironments including granulomas specific to CD and areas of epithelial tissue damage and lymphoid aggregates found in both CD and UC. Upon investigating the inflammatory landscape of CD and UC pretreatment, we discern both pro-remission and pro-inflammatory cellular mediators that are associated with remission outcomes. Pro-remission encompasses specific epithelial and myeloid factors. Conversely, increased cytokine and chemokine expression in specific monocytes were seen in nonremission subgroups at baseline. Our longitudinal design has allowed us to elucidate persisting cellular drivers of nonremission post-adalimumab. In CD, we found a prominent role for specific myeloid autocrine signalling and CD4^+^ T cell-myeloid interactions. In UC, we dissected the multi-cellular nature of nonremission demonstrating the increase of pDCs, multi-compartmental interferon signalling, distinct fibroblast-derived recruitment signatures, specific T helper cell responses and IgG-skewed plasmablasts. Extending the study to RA through the generation of a synovial meta-atlas comprising 520,603 cells reveals a shared TNF pathway expression pattern in CD, UC and RA, as well IFN signalling associated with a lymphoid pathotype. Our therapeutic atlas informs drug positioning across IMIDs and suggests a rationale for the use of JAK and p19 inhibition following anti-TNF resistance. DC, dendritic cell; pCD8T, CD8^+^ T cell/NK GEP; pFP, fibroblast/pericyte GEP; pM: myeloid cell GEP; Th, CD4^+^ helper T cell; Tfh, CD4^+^ follicular helper T cell; Tph, CD4^+^ peripheral helper T cell.

Here, we have profiled intestinal tissues at single-cell resolution in CD and UC, before and after administration of the most widely used biologic, adalimumab. This resource represents the largest longitudinal, therapeutic scRNA-seq atlas to date, comprising ~1 million cells from 216 samples across 41 individuals (Extended Data Fig. 10). This atlas will aid patient stratification and drug discovery efforts in the IMID research community.

The state of the inflammatory landscape at baseline, its longitudinal evolution and its association with adalimumab outcomes have not been previously characterised at single-cell resolution for adult CD and UC. Indeed, prior studies have identified the need for longitudinal cohorts^51^. Previously, signatures proposed to be associated with anti-TNF nonresponse were projected from bulk transcriptomics^8,13,14,28^. Gut bulk transcriptomics may reflect overall cell abundance rather than changes within individual cell populations. In our prospectively recruited IBD cohort with comparable inflammation at baseline, we systematically identified cell states associated with remission/nonremission. The selection of remission as an endpoint, rather than response, is consistent with the clinical treatment goal of mucosal healing^52^.

We explored the shared and distinct drivers of inflammation in CD and UC. Although clinically disparate entities, bulk RNA-seq studies have had limited ability to distinguish them^30^. A CyTOF investigation of immune cells identified differences in cytokine-producing T cells and myeloid cells between CD and UC^53^. We detected Th1 expansion as a hallmark of inflammation in CD but not UC. Markedly increased IgG^+^ plasma cells and plasmablasts were observed in UC, as recently reported^8^. This expansion was also observed, to a lesser degree, in CD. Distinctions between these diseases extended to the epithelium, as the *PLCG2*^hi^ enterocyte was specifically increased in inflamed CD.

We then mapped scRNA-seq-derived GEPs to cellular neighbourhoods in IBD using multiplexed imaging and spatial transcriptomics. IFN-response hubs were profiled using protein markers CXCL9 and CCL19 corresponding to GEPs pM14 and pFP11. pM14 (CXCL9^+^) was present in CD14^+^ CD40^hi^ CD11c^+^ monocyte-derived DCs localising to distinct spatial niches: (1) co-occurrence with CCL19^+^ stromal cells (pFP11) in T cell aggregates and (2) areas of epithelial damage. CCL19^+^ fibroblasts and associated IFN signalling have been described in multiple IMIDs including RA^49^. In UC, pFP11 was strongly correlated with pCD4T15. This GEP is expressed in Th1 and Th1/17 cells, which could be the IFNγ source in this niche. Th1/17 cells have also been implicated in granuloma formation in sarcoidosis-affected skin^46^. In CD, pCD4T15 correlated with pM04, which shares features with granuloma-associated macrophages.

In regions of epithelial damage, neutrophil-attractant fibroblasts are present^28^. These cells were represented by pFP01. This GEP was in the same hub as pCD8T11. pCD8T11 was highly expressed in CD8^+^ *FGFBP2*^+^ T cells, demarcated by *GZMB*. GZMB^+^ CD8A^+^ T cells localised to areas of epithelial damage along with S100A9^+^ MPO^+^ CD66B^+^ neutrophil aggregates and CXCL9^+^ monocyte-derived DCs. As CD8^+^ *FGFBP2*^+^ T cells potently express *IFNG*, they may drive IFN-response in monocyte-derived DCs in this niche.

In the myeloid compartment, GEPs enriched in S100A8/9^hi^ *TNF*^hi^ *IL6*^+^ monocytes belonged to ‘tissue damage’ hubs in CD and UC. Interestingly, although abundance of these monocytes did not vary between remission/nonremission samples at baseline, their transcriptomic features did differ. In nonremission, these monocytes exhibited higher chemokine (*CXCL3*) and cytokine expression (*IL18*). In UC remission, we also found higher expression of the inhibitory receptor *CD300A* at baseline.

Following treatment in CD nonremission, a pro-inflammatory myeloid autocrine loop including IL-1 signalling was detected. We previously described an IL-1-dependent stromal-neutrophil axis in anti-TNF nonresponse in IBD^28^. Consistent with this, there was increased expression of *THY1*, *FAP*, *CXCL5* and *CXCL6* in subepithelial and lamina propria fibroblasts in UC nonremission. We did not capture neutrophils in our dataset, which is a well-recognised caveat of using frozen tissue in 10X single-cell experiments, however, this fibroblast signature was indicative of neutrophil chemoattraction. In *C3*^hi^ *RSPO3*^+^ fibroblasts we saw increased *CCL19* expression indicative of lymphocyte infiltration. Another feature of UC nonremission was increased expression of checkpoint genes across several Th cell states.

Despite longstanding interest in understanding nonresponse to anti-TNF, investigation of the cellular correlates of anti-TNF response has been limited^14,54^. In our study, we move beyond the concept of response merely being associated with reduced inflammation or with the absence of a nonresponse driver. We observed for the first time, that the frequency of *TREM2*-expressing C1Q^hi^ *IL1B*^lo^ macrophages at baseline was associated with remission in CD. *TREM2*-expressing macrophages have been associated with regulating synovial inflammation^18^ and regulatory macrophages have been implicated in IBD anti-TNF efficacy^55–57^.

We also found baseline differences in the epithelium between remission/nonremission groups. Projection of a bulk RNA-seq-derived anti-TNF sensitivity signature has previously been detected in UC epithelium^14^. In a novel observation, colonic goblet cells, specifically, were quantitatively and qualitatively distinct at baseline between remission/nonremission in UC and CD. Interestingly, colonic CD and UC remission groups had higher baseline expression of MHC class I and II and IFN-response genes across multiple epithelial cell states. Recent murine studies report that epithelial MHC class II-dependent antigen presentation limits inflammatory damage^58^.

IFNs are pleiotropic cytokines that drive inflammation but also epithelial regeneration^59,60^. At baseline, pro-inflammatory IFN-responsive hubs mapped to T cell aggregates and tissue damage areas. In CD, IFN-related genes in S100A8/9^hi^ *TNF*^hi^ *IL6*^+^ monocytes were associated with nonremission. Conversely, increased epithelial expression of these genes at baseline associated with remission. Longitudinally in UC remission, there was diminished type I and II IFN-response following successful resolution of inflammation. In nonremission however, IFN-response was increased in the epithelial, immune and stromal compartments, accompanied by pDC expansion. pDC enrichment was previously observed in children with UC who went on to require colectomy^61^. Interestingly, pDC-derived type I IFN may contribute to paradoxical psoriasis following anti-TNF^62^. Whilst IFN-response may be protective in re-establishing epithelial homeostasis in remission, it may be pathogenic in other cell types in adalimumab nonremission. Although not efficacious for all patients, JAK1 or p19 inhibition, which modulate IFN pathways, may be effective in those anti-TNF nonresponders^63–67^ for whom clinical benefit outweighs infection-associated safety risks^66,68^.

The amenability of RA to anti-TNF therapy led us to compare across organ systems. We found analogous TNF pathway gene expression in inflamed gut and synovium. As for IBD, for RA we also detected an IFN-response hub. This hub was enriched in the RA lymphoid pathotype.

Our longitudinal profiling strategy is a starting point to capture dynamic, cellular-level IMID evolution. A limitation was the disparity in sampling time post-treatment. All patients were sampled at least 8 weeks after exposure to adalimumab, but sampling varied up to 1.5 years after therapy because of the COVID-19 pandemic. However, all patients were on therapy at the post-treatment sampling timepoint. Although we used multiplexed imaging to validate inflammatory hubs, and flow cytometry for TNF pathway components, most of our findings are derived from RNA-level data that require quantitative assaying at the protein level. Future studies could also explore patients treated with other anti-TNF agents (for example, infliximab).

With the advent of biosimilars and the plethora of available advanced therapies, characterising cellular associations of treatment outcome to rationalise drug positioning and discovery strategies is imperative^69,70^. Therefore, we examined the cellular basis of inflammation and drug response in CD, UC, and RA. As the most used first-line biologic in adults, our in vivo adalimumab perturbation atlas serves as a foundation for investigating other existing and emerging therapies across IMIDs.

## Methods

Sample size was not predetermined, and patients were not randomised for this observational study.

### Patient cohorts and ethics

Biologic-naïve IBD patients to be escalated to adalimumab were recruited at the John Radcliffe Hospital (Oxford) IBD outpatient clinic. Biopsies were collected (IBD Cohort 09/H1204/30)/(GI Ethics 16/YH/0247), Yorkshire & The Humber - Sheffield Research Ethics Committee) from terminal ileum, ascending colon, descending colon and/or rectum (colonoscopy) or the descending colon, sigmoid and/or rectum (flexible sigmoidoscopy). Clinical history and examination were undertaken to determine disease activity (HBI for CD and SSCAI for UC). Endoscopic (UCEIS for UC, and presence and absence of ulceration for CD) and histologic readouts (Nancy index) were collected. During follow-up, serum trough adalimumab levels were taken to exclude antibody-mediated therapy failure.

Patients with clinically diagnosed RA were recruited and followed up in an observational standard-of-care cohort (South Birmingham Research Ethics Committee: 14/WM/1109). Serial synovial biopsies were taken from biologic-naïve patients under nested ethics (West Midlands Black Country Research Ethics Committee: 07/H1203/57). Patients with RA with a Disease Activity Score-28-ESR score of $\ge$5.1 and active inflammation in at least one biopsiable joint (according to ACR/EULAR 2010 criteria) underwent ultrasound-guided synovial biopsy. Four to six synovial fragments were obtained per small joint and six to eight fragments per large joint. Clinical assessments were undertaken at time of biopsy. Patients were rebiopsied in the same joint after treatment with adalimumab, subject to patient consent and welfare.

### Obtaining samples and preparation of samples for scRNA-seq

All gut tissue samples were obtained in RPMI 1640 Medium (Gibco) on ice and processed within 2 h of the procedure. Sample processing was performed under sterile conditions. Samples were gently washed with 1X PBS, finely macerated with a scalpel and cryopreserved with CryoStore CS10 Cell Freezing Medium (Sigma-Aldrich) and stored in liquid nitrogen. Samples for histology were placed into formalin for paraffin embedding. Synovial tissue was minced using scalpels to ensure fragments were <1 mm in diameter and cryopreserved with CS10.

Peripheral blood (20 ml) was collected by venipuncture from patients with IBD and peripheral blood mononuclear cells (PBMCs) were isolated using Lymphoprep (Stemcell Technologies) gradient. Samples were cryopreserved in10% DMSO/90% foetal bovine serum.

### 10X Genomics scRNA-seq library preparation, tissue dissociation and sequencing

Gut and synovial tissue samples and PBMCs were thawed into warm IMDM media with 10% foetal bovine serum and washed. Gut samples were EDTA-treated predigestion with rotation for 15 min to remove dead/damaged epithelial cells, and then dissociated enzymatically with Liberase TM and DNase into a single-cell suspension with rotation. Thawed synovial samples were digested in Liberase TL and DNase in warm media for 30 min, with agitation.

All cell samples were strained and washed twice with PBS with 0.4% bovine serum albumin (BSA). Live cells were counted using acridine orange/propidium iodide and $\le \,$10,000 cells were loaded per 10X Chromium channel. The GEX 3′ V3 protocol was followed.

### scRNA-seq pre-processing and quality control filtering

Cell Ranger v3.1.0 was used to align reads to reference (GRCh38-3.0.0) and generate feature-barcode matrices from the Chromium single-cell RNA-seq output. Panpipes was used to generate anndata objects following quality control, and batch correction^71^. Filtering steps for high-quality single cells included removal of: doublets using Scrublet^72^, cells expressing <500 genes and cells with mitochondrial gene count percentage >60%. Cells with high mitochondrial content were not overrepresented in inflamed samples above or below the mitochondrial cut-off. Genes that were detected in less than three cells were removed.

### Selection of variable genes, dimensionality reduction, clustering and annotation

UMI counts were normalised by total UMI number per cell and converted to transcripts-per-10,000. Data were log-normalised. Highly variable genes were selected, following which T cell receptor, immunoglobulin and HLA genes were removed. Data were scaled prior to PCA. For gut samples, BBKNN was used for sample batch correction^73^. Leiden clustering was applied to derive broad cell populations for the gut, synovium and PBMC samples. In the synovium, harmony was used to integrate across samples and study of origin^74^. For PBMCs, Vireo was used to demultiplex samples^75^. Harmony was used to integrate across samples and multiplexed sample pool.

Broad cell populations were subclustered with tailored PCAs and n_neighbors in addition to harmony for batch correction. Where individual cell clusters in partitioned datasets demonstrated biological anomalies (for example specific RNA contamination), cells were removed from the analysis. Wilcoxon rank-sum test was used to conduct differential expression between clusters to derive marker genes. False discovery rate (FDR)-adjusted *P* value < 0.05 was considered significant for marker genes and all other analyses unless otherwise specified. Clusters typified by very high mitochondrial content were excluded.

### Derivation of the inflammation score

The inflammation score is a composite gene score. We identified a list of genes differentially expressed between histologically inflamed (as per Nancy index) IBD resections to noninflamed/non-IBD gut tissue following multiple comparison correction using DESeq2 (Supplementary Table 4)^28,76^. Data derived from TAURUS were pseudobulked (sum) at the sample level. We then used this gene list as a gene signature and applied the *enrichIt* function from the *escape* package^77^. The score was scaled between 0–10, resulting in a vector representing enrichment of the inflammation score per sample. The highest inflammation score in the healthy samples was selected as a heuristic inflammation score cut-off.

### Remission criteria

For CD, remission was defined as two out of three: HBI < 5, no macroscopic ulcers, Nancy ≤ 1 at follow-up. For UC, remission was defined as two out of three: SSCAI ≤ 2, UCEIS ≤ 1, Nancy ≤ 1 at follow-up. Escalation to another advanced biologic agent because of uncontrolled disease activity was automatically considered as nonremission. For RA, we used a EULAR good or moderate response to define binary response^78^.

### Differential abundance analysis

PCA association testing was utilised to investigate influence of covariates on cell abundance (Supplementary Table 10)^79^. PCs cumulatively explaining ≤ 90% of variation were tested. Differential abundance was performed using MASC, adjusting for age, sex, treatment (for inflamed vs noninflamed analysis only), site, disease duration, percent of mitochondrial genes, and a nested random effects design, (1| donor/sample) to account for multiple samples per patient^80,81^. Differential abundance was conducted as follows:

i. To detect cell state-specific changes in inflammation, comparison across CD and UC, remission outcome associations at baseline and effect of treatment, cell state abundance was analysed as a proportion of the ‘low’ resolution category.
ii. To detect compartment-specific associations with remission outcomes at baseline and changes following treatment across remission subgroups, compartment abundance was analysed as a proportion of the entire sample.

### Ligand-receptor analysis

MultiNicheNetr was used for differential ligand-receptor analysis, including comparisons of inflamed CD and UC (pretreatment samples only), and examination of baseline differences between remission/nonremission groups in CD and UC^82^. Multifactorial analysis was conducted separately by disease to understand differences in remission groups longitudinally following treatment, using a combination of Remission and Treatment terms. For all comparisons: $\ge$ 10 cells per cell type per sample, and non-zero gene expression value in $\ge$ 5% of cells per sample were required. Statistical *P* values were used, and empirical_pval was FALSE. Default thresholds for logFC (0.5) and *P* value threshold (0.05) were used. P_val_adj was TRUE. Default prioritisation criteria were used.

### PROGENy analysis

To quantify TNF signalling, we employed PROGENy^43^. Linear mixed effects model using the *lmer* function as part of the *lmerTest* package was used to test for association between TNF signalling scores pre-/post-adalimumab with the patient variable accounted as random effects.

### RNAscope

The RNAscope Fluorescent Reagent Kit v2 Assay was used (Advanced Cell Diagnostics). Tissue sections were baked for 1.5 h (60 °C) and dehydrated in ethanol, followed by antigen retrieval and protease treatment. Probes for target genes were hybridised for 2 h (40 °C), washed, and hybridised with target-binding amplifiers. Hybridisation with negative control probes was performed in parallel. The final step of the first hybridisation round attached fluorophores to target genes. Sections were then counterstained with DAPI for 2 min, mounted and coverslipped. Sections were imaged using a 20X objective on an IN Cell Analyzer 2500HS and Cell DIVE (Leica Microsystems).

### scRNA-seq differential expression and pathway analysis

Compartment-level pseudobulked profiles were generated for differential expression analysis. Ileum-colon and pairwise intracolon comparisons were performed using *limma-voom* with *duplicateCorrelation* to account for multiple samples per patient^83^. Linear model was fit using *lmFit*, and moderated t-statistics as well as associated *P* values were generated using the *ebayes* function. For intracolon comparisons, pairwise statistical tests were only conducted for genes reaching adjusted *P* value < 0.05 on the group likelihood ratio test.

PCA association testing was used to investigate influence of covariates on gene expression (Supplementary Table 10)^79^. PCs cumulatively explaining ≤ 80% of variation were tested. MAST was used to compare noninflamed to inflamed samples from the ileum and colon, respectively, in CD and noninflamed to inflamed samples in UC^84^. To longitudinally profile cell state changes, we applied MAST to paired samples (samples from the same region in the same patient before and after treatment). Sample pairs were required to have $\ge \,$1 sample inflamed for inclusion in this analysis. Baseline analyses comparing remission to nonremission outcome only used inflamed samples at baseline from these sample pairs. Genes expressed in $\ge \,$10% of a cell state were tested for differential expression. Covariates included, age, sex, treatment (for inflamed vs noninflamed analysis only), site, disease duration, number of genes detected, and a nested random effects design, (1| donor/sample) to account for multiple samples per patient. For longitudinal analyses, an interaction term of treatment (pre/post) by Remission (Remission/nonremission) was used. Other parameters included method = ‘glmer’, with ebayes=FALSE, and nAGQ=0.

GSEA was run using *ClusterProfiler* with fgseaMultilevel algorithm for MsigDB (version 2023.2) GO:BP, Reactome and Hallmark gene signatures^85^. All genes tested for differential expression were used for gsea. Ranking metric used was −log_10_(unadjusted *P* value) *sign(log_2_FC). Only pathways with adjusted *P* value (Benjamini-Hochberg) < 0.05 were considered significant.

### Identification of GEPs by cNMF

cNMF was iteratively applied to broad cell type categories as identified with Leiden clustering. These included B, plasma, CD4^+^ T, CD8^+^ T, myeloid, stromal (fibroblasts and pericytes), myofibroblast, endothelial, colonic epithelial, ileal epithelial, glial and innate lymphoid cells. In the synovium, these categories were B, plasma, T, myeloid and stromal cells.

Briefly, we applied cNMF to a count matrix, *N* (cells) × *M* (genes) to derive two matrices: *k* (GEP) × *M* (genes), and *N* (cells) × *k* (GEP) with the usage of each GEP per cell^45^. Selection of *k* was dependent on several factors including prioritising solutions that were biologically meaningful according to top weighted genes, factorisation stability as determined by silhouette score and minimisation of the Frobenius reconstruction error. Consensus solutions were filtered for outliers through inspection of distances between components and their nearest neighbours by histogram. GEP-associated genes were identified using multiple least squares regression of normalised (z-scored) gene expression against the consensus GEP usage matrix. Overrepresentation analysis for all GEPs was conducted through GOATOOLS with top 150 weighted genes^86^ as input and all genes in the relevant matrix as the gene universe.

### Identification of hubs and calculating NMF transcriptional programme activity

Hubs were identified through analysis of covarying GEPs in inflamed samples for CD and UC separately^87^. Programme activity was calculated for every GEP according to the cell type category of identification. GEP activity was summarised across individual samples^87^. We calculated GEP expression across five quantiles (0.25, 0.5, 0.75, 0.95, 0.99) per sample. Per quantile, a Pearson correlation co-efficient (*R*) was derived for each GEP pair across samples. The correlation was Fisher-transformed and correlation mean was used as a test statistic. We compared *R* against a null distribution derived by permuting sample identity 10,000 times, keeping cell type constant. *P* values were generated by counting how often the permuted *R* value was above and below the true *R* value. Minimum count was scaled by two and designated the *P* value statistic. Multiple comparisons were corrected at Benjamini-Hochberg FDR = 10%. We derived an adjusted *R* value by calculating the difference between mean true *R* values and mean permuted *R* values.

Significant Fisher-transformed associations, *R* (edges) and their constituent GEPs (nodes) were used to create a signed weighted network. Hubs within this network were detected using a module detection algorithm used for signed graphs^88^. This was applied by resolution parameter in the range of 0.001 to 0.2, and tau = 0.2. This method was iteratively applied, and hubs split if they were larger than three nodes and improved modularity of the solution.

### Testing GEP enrichment in inflammation

We calculated the GEP mean activity values at five percentiles (0.25 0.5, 0.75, 0.95, 0.99) per sample. Linear mixed effects model using the *lmer* function as part of the *lmerTest* package was used to test for GEP enrichment in gut inflammation. Association between mean GEP expression and inflammation status was tested with covariates including age, sex, site, disease duration, treatment and random effects term for patient. IBD hubs were deemed inflammatory if >50% of constituent GEPs in a hub were enriched in inflammation. RA hubs were deemed inflammatory if >50% of constituent GEPs in a hub were positively correlated with CD45^+^ cell proportion per sample^49^.

### Projection of GEPs to bulk RNA sequencing and GeoMx data

As above, cNMF yields a *k* (GEP) × *M* (genes) matrix, henceforth referred to as *H*. The gene expression matrix from the relevant bulk RNA sequencing/GeoMx data were subsetted to genes shared with *H*. NMF was initialised with *H* and the gene expression matrix to generate the projected component matrix, *W* (samples × *k*). The NMF implementation used was *sklearn.decomposition.non_negative_factorization*.

### Processing bulk RNA sequencing data from R4RA

FASTQ files generated from the R4RA trial were downloaded from EMBL-EBI (E-MTAB-11611). Files were trimmed to remove low-quality reads using trimgalore (0.6.6) in paired mode and aligned to the human genome (GRCh38, Ensembl release 101) using STAR (2.7.3a). Gene counts were summarised using featureCounts (Subread v2.0.1). Raw counts were RPKM-normalised using edgeR functions *calcNormFactors* (TMM) and *rpkm*.

### Multiplexed imaging using Cell DIVE

#### Slide clearing and blocking

Four-micron-thick formalin-fixed paraffin-embedded (FFPE) gut tissue slides were deparaffinised and rehydrated. Slides were then permeabilised for 10 min in 0.3% Triton X-100 and washed. Antigen retrieval was performed using the NxGen decloaking chamber (Biocare Medical) in boiling pH6 Citrate (Agilent) and pH9 Tris-based antigen retrieval solutions for 20 min each. Tissue slides were blocked in 1X PBS/3% BSA (Merck)/10% donkey serum (Bio-Rad) for 1 h at room temperature (RT). Slides were washed, stained with DAPI, washed again and coverslipped with mounting media (50% glycerol and 4% propyl gallate, Sigma).

#### Scan plan and background acquisition

The GE Cell DIVE system was used to image FFPE slides. A scan plan was acquired at ×10 magnification for region selection, followed by imaging at ×20 to acquire background autofluorescence and generate virtual H&E images. Background imaging was used to subtract autofluorescence from subsequent staining rounds. Slides were de-coverslipped before staining.

#### Staining and bleaching

Multiplexed imaging included staining for protein markers at the following concentrations: CD66B (2 μl ml^−1^), 2.5 μl ml^−1^ (CD208, S100A9), GZMB (3 μl ml^−1^), 5 μl ml^−1^ (CD68, CD3, CCL19, CK8, CD4, CD20, CXCL9, KI67, MPO, CD14, CCR7, CD11C CD40, PD1, MZB1, COL1A1), 10 μl ml^−1^ (CD8A, CXCL13). Each staining round consisted of three antibodies prepared in blocking buffer (PBS, 3% BSA, 10% donkey serum). The initial round used primary antibodies incubated overnight at 4°C followed by washes in 1X PBS and 0.05% Tween20. Secondary antibodies raised in donkey and conjugated to Alexa Fluorophore-488, 555 or 647 (Invitrogen) were then incubated for 1 h (RT). Each subsequent staining round used directly conjugated antibodies with overnight incubation (4°C). Manually conjugated antibodies were BSA-AZIDE-free and conjugated using antibody-labelling kit (Invitrogen). Fluorophores were bleached between staining rounds using NaHCO_3_ (0.1 M, pH 11.2, Sigma) and 3% H_2_O_2_ (Merck). DAPI staining between imaging rounds assisted image registration and alignment. Slides were multiplexed with the next three-marker panel with iterative staining, bleaching and imaging.

#### NanoString GeoMx DSP spatial transcriptomics profiling

Sections of 5 μm were cut from FFPE tissue blocks under RNase-free conditions, placed onto Leica adhesive microscopic slides and baked overnight (60 °C). Manual slide preparation was conducted according to NanoString’s protocol. Slides were deparaffinized and rehydrated. Target retrieval was performed using IHC Antigen Retrieval Solution (eBioscience) for 20 min (100 °C), followed by 15 min (37 °C) in 1 μg ml^−1^ Ambion Proteinase K (ThermoFisher Scientific). After retrieval, slides were fixed in 10% neutral buffered formalin and washed. Samples were UV light-treated (405 nm, 24 h) to quench background autofluorescence. Next, slides were incubated with human Whole Transcriptome Atlas probes (NanoString) for 16 h (37 °C).

Slides were washed in formamide-SCC buffer before tissue blocking and immunofluorescent staining in Buffer W (NanoString) with 1% Fc-Receptor block (Miltenyi)/5% donkey serum (Jackson ImmunoResearch). Sections were incubated in blocking buffer (RT) with 1 μg ml^−1^ anti-CD68 (SantaCruz, mouse KP1) and 5 μg ml^−1^ anti-CD3 (Abcam, rabbit SP162) for 1 h; followed by 1:1,000 anti-mouse-AF647 (Jackson ImmunoResearch, 115-605-006), 1:1,000 anti-rabbit-Cy3 (Jackson Immuno Research, 111-165-006), 1:40 CD45-AF594 (Nanostring) and 1:20,000 Sytox Green (Invitrogen, S7020) for 1 h.

Slides were imaged with the Nanosting GeoMx Digital Spatial Profiler with manual selection of regions of interest, from which oligonucleotide probes were collected. For library generation, samples were subjected to PCR using i5 and i7 dual indexing primers (Nanostring) before pooling and purification using AMPure XP beads (Beckman Coulter). Library QC was done using Qubit (ThermoFisher Scientific) and TapeStation (Agilent). Resulting libraries were sequenced on the Illumina NovaSeq platform using 150-bp paired-end sequencing.

FASTQ files generated were converted into DCC files using the GeoMxNGSPipeline (version 2.0.0.16). Regions with ≥ 1% of probe target detection were selected. Only genes detected in ≥ 10% of samples were retained. Data were Q3-normalised and log_2_-transformed. The *mixedModelDE* function was used to test for association with lymphoid aggregate/lamina propria with a random effect term for slide.

#### Flow cytometry

PBMCs were stained with antibodies at 2 μg ml^−1^, including: mouse anti-human TNFR1-APC mAb (clone W15099A, BioLegend); rat anti-human TNFR2-PE mAb (clone hTNFR-M1, BD Biosciences); mouse anti-human TNF-alpha (clone MAb11, BioLegend). After washing, cells were fixed for 20 min in 4% paraformaldehyde (RT) or fixed/permeabilised according to manufacturers’ instructions (BD Biosciences Cytofix/Cytoperm). For intracellular staining, antibodies were incubated in permeabilisation buffer for 30 min (RT). Stained cells were acquired on a BD LSRII.

### Reporting summary

Further information on research design is available in the Nature Portfolio Reporting Summary linked to this article.

## Online content

Any methods, additional references, Nature Portfolio reporting summaries, source data, extended data, supplementary information, acknowledgements, peer review information; details of author contributions and competing interests; and statements of data and code availability are available at 10.1038/s41590-024-01994-8.

## Supplementary information

Supplementary Information Supplementary Figures 1–5.

Reporting Summary

Supplementary Table 1 Study cohort summary and metadata.

Supplementary Table 2 Marker genes for gut cell states.

Supplementary Table 3 Differential expression of ileum compared to colon, and intracolon sites.

Supplementary Table 4 Differential abundance and expression of inflamed vs noninflamed in CD and UC.

Supplementary Table 5 GEPs and associated enrichment analysis in inflammation.

Supplementary Table 6 Baseline and longitudinal differential abundance analysis in CD and UC.

Supplementary Table 7 Baseline differential expression comparing remission outcomes in CD and UC.

Supplementary Table 8 Longitudinal differential expression in CD and UC.

Supplementary Table 9 RA-related analyses.

Supplementary Table 10 Principal component analysis of cell abundance and gene expression.

## Data Availability

All raw and processed data are available via Zenodo (10.5281/zenodo.13768607).

## Code availability

All source code will be available on GitHub: https://github.com/DendrouLab/TAURUS_paper. Supplementary information is available for this paper.
