# Supplementary material for: A longitudinal single-cell atlas of anti-tumour necrosis factor treatment in inflammatory bowel disease
Source: Nat Immunol. 2024 Oct 22;25(11):2152–65. doi: 10.1038/s41590-024-01994-8 (PMC11519010; doi:10.1038/s41590-024-01994-8)
Supplement: Supplementary file 1 — Supplementary Figures 1–5. [file 41590_2024_1994_MOESM1_ESM.pdf]

nature immunology

Supplementary information

https://doi.org/10.1038/s41590-024-01994-8

A longitudinal single-cell atlas of anti-tumour necrosis factor treatment in inflammatory bowel disease

In the format provided by the authors and unedited

Supplementary Figures for:

A longitudinal single-cell atlas of anti-tumour necrosis factor treatment in inflammatory bowel disease

Tom Thomas[1,2,3], Matthias Friedrich[1,3*], Charlotte Rich-Griffin[2*], Mathilde Pohin[1*], Devika Agarwal[1*], Julia Pakpoor[1,2,3], Carl Lee[1], Ruchi Tandon[4], Aniko Rendek[5], Dominik Aschenbrenner[3], Ashwin Jainarayanan[1], Alexandru Voda[1], Jacqueline HY Siu[1], Raphael Sanches-Peres[1], Eloise Nee[1], Dharshan Sathananthan[6,7], Dylan Kotliar[8,9], Peter Todd[2], Maria Kiourlappou[2], Lisa Gartner[3], Nicholas Ilott[1], Fadi Issa[10], Joanna Hester[10], Jason Turner[11], Saba Nayar[11,12,13], Jonas Mackerodt[1], IBD Cohort Investigators, AMP RA investigators, Fan Zhang[8,9,14], Anna Jonsson[8,9], Michael Brenner[8,9], Soumya Raychaudhuri[8,9], Ruth Kulicke[15], Danielle Ramsdell[15], Nicolas Stransky[15], Ray Pagliarini[15], Piotr Bielecki[15], Noah Spies[15], Brian Marsden[2], Stephen Taylor[2], Allon Wagner[16,17], Paul Klenerman[3], Alissa Walsh[3], Mark Coles[1], Luke Jostins-Dean[1], Fiona M Powrie[1], Andrew Filer[11,12,13], Simon Travis[1,3,18#], Holm H Uhlig[3,18, 19#], Calliope A Dendrou[1,2,18#], Christopher D Buckley[1,3,11,18#]

[*] These authors contributed equally [#] These authors jointly supervised this work

[1]Kennedy Institute of Rheumatology, Old Road Campus, University of Oxford, Oxford, UK

[2]Centre for Human Genetics, Old Road Campus, University of Oxford, Oxford, UK

[3]Translational Gastroenterology & Liver Unit, John Radcliffe Hospital, Headington, Oxford, UK

[4]University College London Hospitals NHS Foundation Trust, London, UK

[5]Oxford University Hospitals NHS Foundation Trust, Oxford, UK

[6]University of Adelaide, Adelaide, Australia

[7]Lyell McEwin Hospital, Haydown Road, Elizabeth Vale, Australia

[8]Broad Institute of MIT and Harvard, Cambridge, Massachusetts, USA

[9]Department of Medicine, Brigham and Women's Hospital, Boston, Massachusetts, USA

[10] Nuffield Department of Surgical Sciences, University of Oxford, Oxford, United Kingdom

[11]Rheumatology Research Group, Institute of Inflammation and Ageing, University of Birmingham, UK

[12]National Institute for Health Research (NIHR) Birmingham Biomedical Research Centre and NIHR Clinical Research Facility, University Hospitals Birmingham NHS Foundation Trust, Birmingham, UK

[13]Birmingham Tissue Analytics, Institute of Translational Medicine, University of Birmingham, UK

[14]Center for Health AI, University of Colorado Anschutz, Colorado, USA

[15]Celsius Therapeutics, 399 Binney St, Cambridge, Massachusetts, USA

[16]Department of Electrical Engineering and Computer Science, University of California, Berkeley, California, USA

[17]The Center for Computational Biology, University of California, Berkeley, California, USA

[18]NIHR Oxford Biomedical Research Centre, Oxford, UK

[19] Department of Paediatrics, University of Oxford, Oxford, UK

Supplementary Figure List:

Supplementary Figure 1| Top weighted genes of GEPs in the gut.

Supplementary Figure 2| Longitudinal gene expression following adalimumab in CD and UC.

Supplementary Figure 3| Therapeutic atlas for CD.

Supplementary Figure 4| Therapeutic atlas for UC.

Supplementary Figure 5| Top weighted genes of GEPs in the synovium.

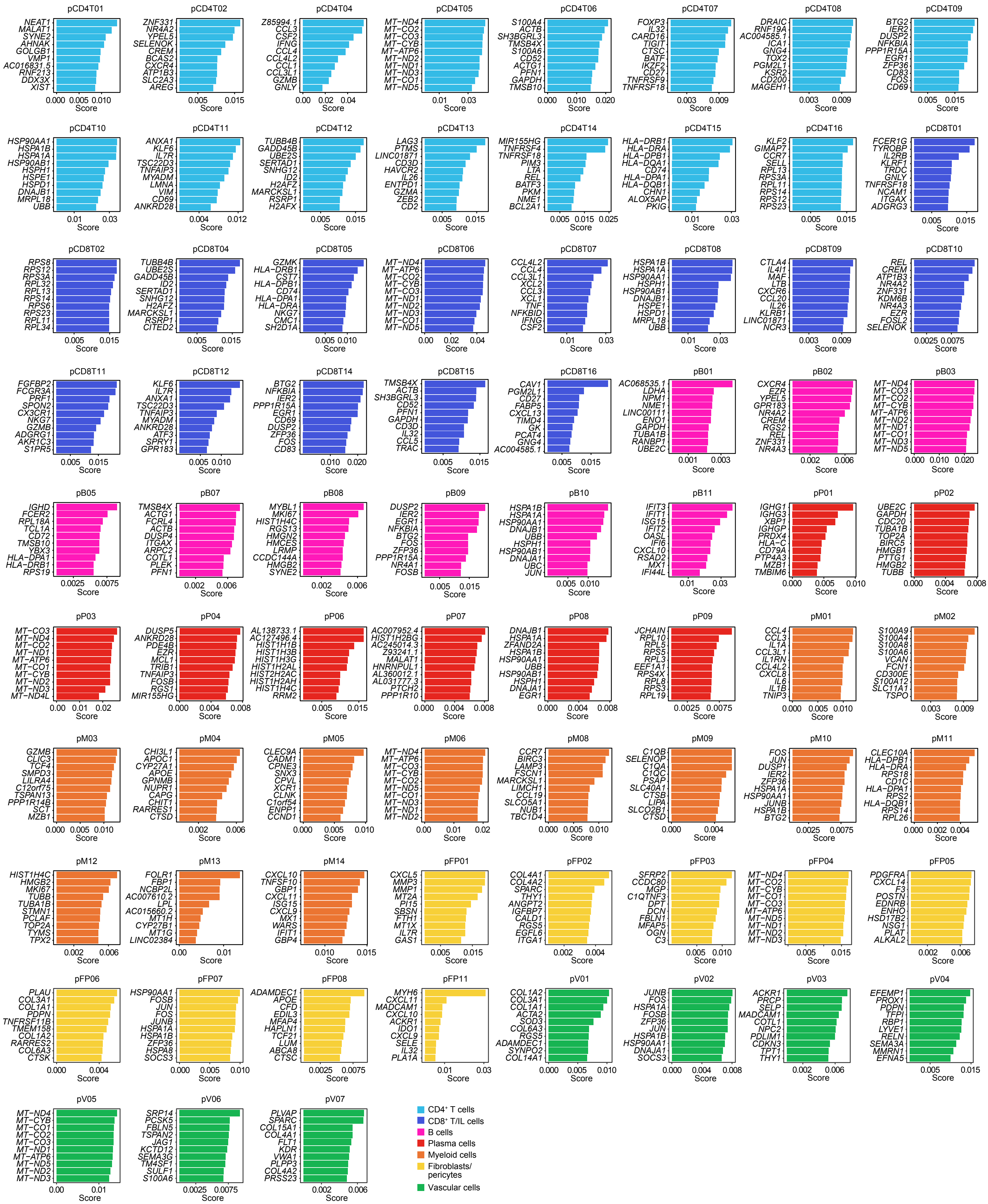

pCD4T01: NEAT1, MALAT1, SYNE2, AHNAK, GOLGB1, VMP1, AC016831.5, RNF213, DDX3X, XIST
pCD4T02: ZNF331, NR4A2, YPEL5, SELENOK, CREM, BCAS2, CXCR4, ATP1B3, SLC2A3, AREG
pCD4T04: Z85994.1, CCL3, CSF2, IFNG, CCL4, CCL4L2, CCL1, CCL3L1, GZMB, GNLY
pCD4T05: MT−ND4, MT−CO2, MT−CO3, MT−CYB, MT−ATP6, MT−ND2, MT−ND1, MT−ND3, MT−CO1, MT−ND5
pCD4T06: S100A4, ACTB, SH3BGRL3, TMSB4X, S100A6, CD52, ACTG1, PFN1, GAPDH, TMSB10
pCD4T07: FOXP3, IL32, CARD16, TIGIT, CTSC, BATF, IKZF2, CD27, TNFRSF9, TNFRSF18
pCD4T08: DRAIC, RNF19A, AC004585.1, ICA1, GNG4, TOX2, PGM2L1, KSR2, CD200, MAGEH1
pCD4T09: BTG2, IER2, DUSP2, NFKBIA, PPP1R15A, EGR1, ZFP36, GNLY, CD83, FOS, CD69
pCD4T10: HSP90AA1, HSPA1B, HSPA1A, HSP90AB1, HSPH1, HSPE1, HSPD1, DNAJB1, MRPL18, UBB
pCD4T11: ANXA1, KLF6, IL7R, TSC22D3, TNFAIP3, MYADM, LMNA, VIM, CD69, ANKRD28
pCD4T12: TUBB4B, GADD45B, UBE2S, SERTAD1, SNHG12, ID2, H2AFZ, MARCKSL1, RSRP1, H2AFX
pCD4T13: LAG3, PTMS, LINC01871, CD3D, HAVCR2, IL26, ENTPD1, GZMA, ZEB2, CD2
pCD4T14: MIR155HG, TNFRSF4, TNFRSF18, PIM3, LTA, REL, BATF3, PKM, NME1, BCL2A1
pCD4T15: HLA−DRB1, HLA−DRA, HLA−DPB1, HLA−DQA1, CD74, HLA−DPA1, HLA−DQB1, CHN1, ALOX5AP, PKIG
pCD4T16: KLF2, GIMAP7, CCR7, SELL, RPL13, RPS3A, RPL11, RPS14, RPS12, RPS23
pCD8T01: FCER1G, TYROBP, IL2RB, KLRF1, TRDC, GNLY, TNFRSF18, NCAM1, ITGAX, ADGRG3
pCD8T02: RPS8, RPS12, RPS3A, RPL32, RPL13, RPS14, RPS6, RPS23, RPL11, RPL34
pCD8T04: TUBB4B, UBE2S, GADD45B, ID2, SERTAD1, SNHG12, H2AFZ, MARCKSL1, RSRP1, CITED2
pCD8T05: GZMK, HLA−DRB1, CST7, HLA−DPB1, CD74, HLA−DPA1, HLA−DRA, NKG7, CMC1, SH2D1A
pCD8T06: MT−ND4, MT−ATP6, MT−CO2, MT−CYB, MT−CO3, MT−ND1, MT−ND2, MT−ND3, MT−CO1, MT−ND5
pCD8T07: CCL4L2, CCL4, CCL3L1, XCL2, CCL3, XCL1, TNF, NFKBID, IFNG, CSF2
pCD8T08: HSPA1B, HSPA1A, HSP90AA1, HSPH1, HSP90AB1, DNAJB1, HSPE1, HSPD1, MRPL18, UBB
pCD8T09: CTLA4, IL4I1, MAF, LTB, CXCR6, CCL20, IL26, KLRB1, LINC01871, NCR3
pCD8T10: REL, CREM, ATP1B3, NR4A2, ZNF331, TRDC, KDM6B, NR4A3, EZR, FOSL2, SELENOK
pCD8T11: FGFBP2, FCGR3A, PRF1, SPON2, CX3CR1, NKG7, GZMB, ADGRG1, AKR1C3, S1PR5
pCD8T12: KLF6, IL7R, ANXA1, TSC22D3, TNFAIP3, MYADM, ANKRD28, ATF3, SPRY1, GPR183
pCD8T14: BTG2, NFKBIA, IER2, PPP1R15A, EGR1, CD69, DUSP2, ZFP36, FOS, CD83
pCD8T15: TMSB4X, ACTB, SH3BGRL3, CD52, PFN1, GAPDH, CD3D, IL32, CCL5, TRAC
pCD8T16: CAV1, PGM2L1, CD27, FABP5, CXCL13, TIMD4, GK, PCAT4, GNG4, AC004585.1
pB01: AC068535.1, LDHA, NPM1, NME1, LINC00111, ENO1, GAPDH, TUBA1B, RANBP1, UBE2C
pB02: CXCR4, EZR, YPEL5, GPR183, NR4A2, CREM, RGS2, REL, ZNF331, NR4A3
pB03: MT−ND4, MT−CO3, MT−CO2, MT−CYB, MT−ATP6, MT−ND2, MT−ND1, MT−CO1, MT−ND3, MT−ND5
pB05: IGHD, FCER2, RPL18A, TCL1A, CD72, TMSB10, YBX3, HLA−DPA1, HLA−DRB1, RPS19
pB07: TMSB4X, ACTG1, FCRL4, ACTB, DUSP4, ITGAX, ARPC2, COTL1, PLEK, PFN1
pB08: MYBL1, MKI67, HIST1H4C, RGS13, HMGN2, HMCES, LRMP, CCDC144A, HMGB2, SYNE2
pB09: DUSP2, IER2, EGR1, NFKBIA, BTG2, FOS, ZFP36, PPP1R15A, NR4A1, FOSB
pB10: HSPA1B, HSPA1A, HSP90AA1, DNAJB1, UBB, HSPH1, HSP90AB1, DNAJA1, UBC, JUN
pB11: IFIT3, IFIT1, ISG15, IFIT2, OASL, IFI6, CXCL10, RSAD2, MX1, IFI44L
pP01: IGHG1, IGHG3, XBP1, IGHGP, PRDX4, HLA−C, CD79A, PTP4A3, MZB1, TMBIM6
pP02: UBE2C, GAPDH, CDC20, TUBA1B, TOP2A, BIRC5, HMGB1, PTTG1, HMGB2, TUBB
pP03: MT−CO3, MT−ND4, MT−CO2, MT−ND1, MT−ATP6, MT−CO1, MT−CYB, MT−ND2, MT−ND3, MT−ND4L
pP04: DUSP5, ANKRD28, PDE4B, EZR, MCL1, TRIB1, TNFAIP3, FOSB, RGS1, MIR155HG
pP06: AL138733.1, AC127496.4, HIST1H1B, HIST1H3B, HIST1H3G, HIST1H2AL, HIST2H2AC, HIST1H2AH, HIST1H4C, RRM2
pP07: AC007952.4, HIST1H2BG, AC245014.3, Z93241.1, MALAT1, HNRNPUL1, AL360012.1, AL031777.3, PTCH2, PPP1R10
pP08: DNAJB1, HSPA1A, ZFAND2A, HSPA1B, HSP90AA1, UBB, HSP90AB1, HSPH1, DNAJA1, EGR1
pP09: JCHAIN, RPL10, RPL5, RPS5, RPL3, EEF1A1, RPS4X, RPL8, RPS3, RPL19
pM01: CCL4, CCL3, IL1A, CCL3L1, IL1RN, CCL4L2, CXCL8, IL6, IL1B, TNIP3
pM02: S100A9, S100A4, S100A8, S100A6, VCAN, FCN1, CD300E, S100A12, SLC11A1, TSPO
pM03: GZMB, CLIC3, TCF4, SMPD3, LILRA4, C12orf75, TSPAN13, PPP1R14B, SCT, MZB1
pM04: CHI3L1, APOC1, CYP27A1, APOE, GPNMB, NUPR1, CAPG, CHIT1, RARRES1, CTSD
pM05: CLEC9A, CADM1, CPNE3, SNX3, CPVL, CLNK, C1orf54, ENPP1, CCND1
pM06: MT−ND4, MT−ATP6, MT−CO3, MT−CYB, MT−CO2, MT−ND5, MT−CO1, MT−ND3, MT−ND1, MT−ND2
pM08: CCR7, BIRC3, LAMP3, FSCN1, MARCKSL1, LIMCH1, CCL19, SLCO5A1, NUB1, TBC1D4
pM09: C1QB, SELENOP, C1QA, C1QC, PSAP, SLC40A1, CTSB, LIPA, SLCO2B1, CTSD
pM10: FOS, JUN, DUSP1, IER2, ZFP36, HSPA1A, HSP90AA1, JUNB, HSPA1B, BTG2
pM11: CLEC10A, HLA−DPB1, HLA−DRA, RPS18, CD1C, HLA−DPA1, RPS2, HLA−DQB1, RPS14, RPL26
pM12: HIST1H4C, HMGB2, MKI67, TUBB, TUBA1B, STMN1, PCLAF, TOP2A, TYMS, TPX2
pM13: FOLR1, FBP1, NCBP2L, AC007610.2, LPL, AC015660.2, MT1H, CYP27B1, MT1G, LINC02384
pM14: CXCL10, TNFSF10, GBP1, CXCL11, ISG15, CXCL9, MX1, WARS, IFIT1, GBP4
pFP01: CXCL5, MMP3, MMP1, MT2A, PI15, SBSN, FTH1, MT1X, IL7R, GAS1
pFP02: COL4A1, COL4A2, SPARC, THY1, ANGPT2, IGFBP7, CALD1, RGS5, EGFL6, ITGA1
pFP03: SFRP2, CCDC80, MGP, C1QTNF3, DPT, DCN, FBLN1, MFAP5, OGN, C3
pFP04: MT−ND4, MT−CO2, MT−CYB, MT−CO1, MT−CO3, MT−ATP6, MT−ND5, MT−ND1, MT−ND2, MT−ND3
pFP05: PDGFRA, CXCL14, F3, POSTN, EDNRB, ENHO, HSD17B2, NSG1, PLAT, ALKAL2
pFP06: PLAU, COL3A1, COL1A1, PDPN, TNFRSF11B, TMEM158, COL1A2, RARRES2, COL6A3, CTSK
pFP07: HSP90AA1, FOSB, JUN, FOS, JUNB, HSPA1A, HSPA1B, ZFP36, HSPA8, SOCS3
pFP08: ADAMDEC1, APOE, CFD, EDIL3, MFAP4, HAPLN1, TCF21, LUM, ABCA8, CTSC
pFP11: MYH6, CXCL11, MADCAM1, CXCL10, ACKR1, IDO1, CXCL9, SELE, IL32, PLA1A
pV01: COL1A2, COL3A1, COL1A1, ACTA2, SOD3, COL6A3, RGS5, ADAMDEC1, SYNPO2, COL14A1
pV02: JUNB, FOS, HSPA1A, FOSB, ZFP36, JUN, HSPA1B, HSP90AA1, DNAJA1, SOCS3
pV03: ACKR1, PRCP, SELP, MADCAM1, COTL1, NPC2, PDLIM1, CDKN3, TPT1, THY1
pV04: EFEMP1, PROX1, PDPN, TFPI, RBP1, LYVE1, RELN, SEMA3A, MMRN1, EFNA5
pV05: MT−ND4, MT−CYB, MT−CO1, MT−CO2, MT−CO3, MT−ND1, MT−ATP6, MT−ND5, MT−ND2, MT−ND3
pV06: SRP14, PCSK5, FBLN5, TSPAN2, JAG1, KCTD12, SEMA3G, TM4SF1, SULF1, S100A6
pV07: PLVAP, SPARC, COL15A1, COL4A1, FLT1, KDR, VWA1, PLPP3, COL4A2, PRSS23
Score
CD4+ T cells
CD8+ T/IL cells
B cells
Plasma cells
Myeloid cells
Fibroblasts/ pericytes
Vascular cells

Supplementary Figure 1| Top weighted genes of GEPs in the gut.

Weighted genes for each gene expression programme (GEP) derived from the gut. cNMF was run separately in: $CD4^+$ T, $CD8^+$ T, B, plasma cells, myeloid cells (monocytes, macrophages and DC), vascular cells, and fibroblasts and pericytes. See **Supplementary Table 5** for full list of weighted genes, results of overrepresentation analysis, and results of enrichment testing of GEPs in inflammation. pB: B cell GEP; pCD4T: $CD4^+$ T cell GEP; pCD8T: $CD8^+$ T cell/NK GEP; pFP: fibroblast and pericyte GEP; pM: myeloid cell GEP; pP: plasma cell GEP; pV: vascular cell GEP.

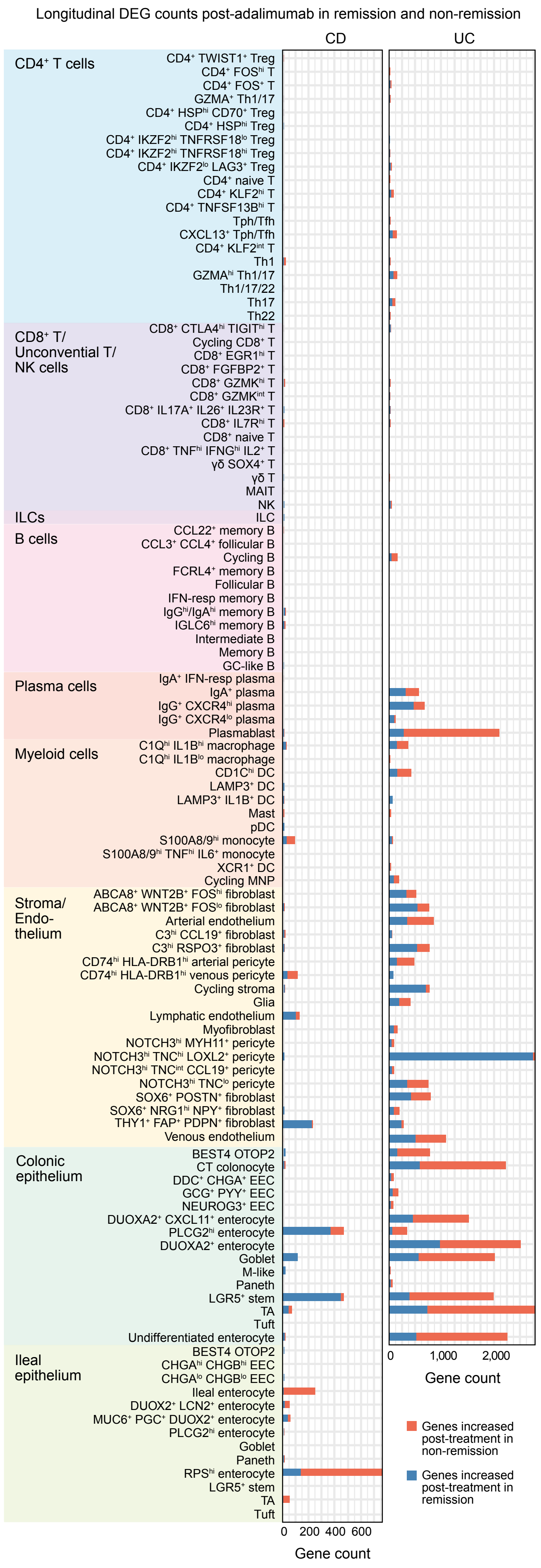

Longitudinal DEG counts post-adalimumab in remission and non-remission
CD
UC
CD4+ T cells
CD4+ TWIST1+ Treg
CD4+ FOShi T
CD4+ FOS+ T
GZMA+ Th1/17
CD4+ HSPhi CD70+ Treg
CD4+ HSPhi Treg
CD4+ IKZF2hi TNFRSF18lo Treg
CD4+ IKZF2hi TNFRSF18hi Treg
CD4+ IKZF2lo LAG3+ Treg
CD4+ naive T
CD4+ KLF2hi T
CD4+ TNFSF13Bhi T
Tph/Tfh
CXCL13+ Tph/Tfh
CD4+ KLF2int T
Th1
GZMAhi Th1/17
Th1/17/22
Th17
Th22
CD8+ T/ Unconventional T/ NK cells
CD8+ CTLA4hi TIGIThi T
Cycling CD8+ T
CD8+ EGR1hi T
CD8+ FGFBP2+ T
CD8+ GZMKhi T
CD8+ GZMKint T
CD8+ IL17A+ IL26+ IL23R+ T
CD8+ IL7Rhi T
CD8+ naive T
CD8+ TNFhi IFNGhi IL2+ T
γδ SOX4+ T
γδ T
MAIT
NK
ILCs
ILC
B cells
CCL22+ memory B
CCL3+ CCL4+ follicular B
Cycling B
FCRL4+ memory B
Follicular B
IFN-resp memory B
IgGhi/IgAhi memory B
IGLC6hi memory B
Intermediate B
Memory B
GC-like B
Plasma cells
IgA+ IFN-resp plasma
IgA+ plasma
IgG+ CXCR4hi plasma
IgG+ CXCR4lo plasma
Plasmablast
Myeloid cells
C1Qhi IL1Bhi macrophage
C1Qhi IL1Blo macrophage
CD1Chi DC
LAMP3+ DC
LAMP3+ IL1B+ DC
Mast
pDC
S100A8/9hi monocyte
S100A8/9hi TNFhi IL6+ monocyte
XCR1+ DC
Cycling MNP
Stroma/ Endo- thelium
ABCA8+ WNT2B+ FOShi fibroblast
ABCA8+ WNT2B+ FOSlo fibroblast
Arterial endothelium
C3hi CCL19+ fibroblast
C3hi RSPO3+ fibroblast
CD74hi HLA-DRB1hi arterial pericyte
CD74hi HLA-DRB1hi venous pericyte
Cycling stroma
Glia
Lymphatic endothelium
Myofibroblast
NOTCH3hi MYH11+ pericyte
NOTCH3hi TNChi LOXL2+ pericyte
NOTCH3hi TNCint CCL19+ pericyte
NOTCH3hi TNClo pericyte
SOX6+ POSTN+ fibroblast
SOX6+ NRG1hi NPY+ fibroblast
THY1+ FAP+ PDPN+ fibroblast
Venous endothelium
Colonic epithelium
BEST4 OTOP2
CT colonocyte
DDC+ CHGA+ EEC
GCG+ PYY+ EEC
NEUROG3+ EEC
DUOXA2+ CXCL11+ enterocyte
PLCG2hi enterocyte
DUOXA2+ enterocyte
Goblet
M-like
Paneth
LGR5+ stem
TA
Tuft
Undifferentiated enterocyte
Ileal epithelium
BEST4 OTOP2
CHGAhi CHGBhi EEC
CHGAlo CHGBlo EEC
Ileal enterocyte
DUOX2+ LCN2+ enterocyte
MUC6+ PGC+ DUOX2+ enterocyte
PLCG2hi enterocyte
Goblet
Paneth
RPShi enterocyte
LGR5+ stem
TA
Tuft
0 200 400 600
Gene count
0 1,000 2,000
Gene count
Genes increased post-treatment in non-remission
Genes increased post-treatment in remission

Supplementary Figure 2| Longitudinal gene expression following adalimumab in CD and UC.

Longitudinal gene expression analysis was conducted separately for CD and UC using MAST on paired samples i.e. (samples from the same region in the same patient before pre- and after post-treatment). Sample pairs were required to have at least one sample inflamed sample for inclusion in this analysis. Sample numbers are presented in main Fig. 4 legend. Only genes expressed in 10% of a cell state were tested for differential expression. Covariates included, age, sex, site, disease duration, number of genes detected, and a nested random effects design, (1| donor/sample) to account for multiple samples per patient. For longitudinal analyses, an interaction term of treatment (pre/post) by remission (remission/non- remission) was used. Stacked barplots show genes increasing in non-remission (post-pre, red), and remission (post-pre, blue) in CD (left) and UC (right). DC, dendritic cell; DEG, differentially expressed gene; EEC, enteroendocrine cell; FC, fold change; fibro, fibroblast; GC, germinal centre; hi, high; IFN-resp, interferon-responsive; ILC, innate lymphoid cell; int, intermediate; lo, low; MAIT, mucosal-associated invariant T; MNP, mononuclear phagocyte; mono, monocyte; NK, natural killer cells; pDC, plasmacytoid dendritic cell; $RPS^{hi,}$ ribosomal protein S-high; TA, transit-amplifying; Tfh, $CD4^+$ follicular helper T cell; Tph, $CD4^+$ peripheral helper T cell; Th, $CD4^+$ T helper cell; Treg, $CD4^+$ regulatory T cell.

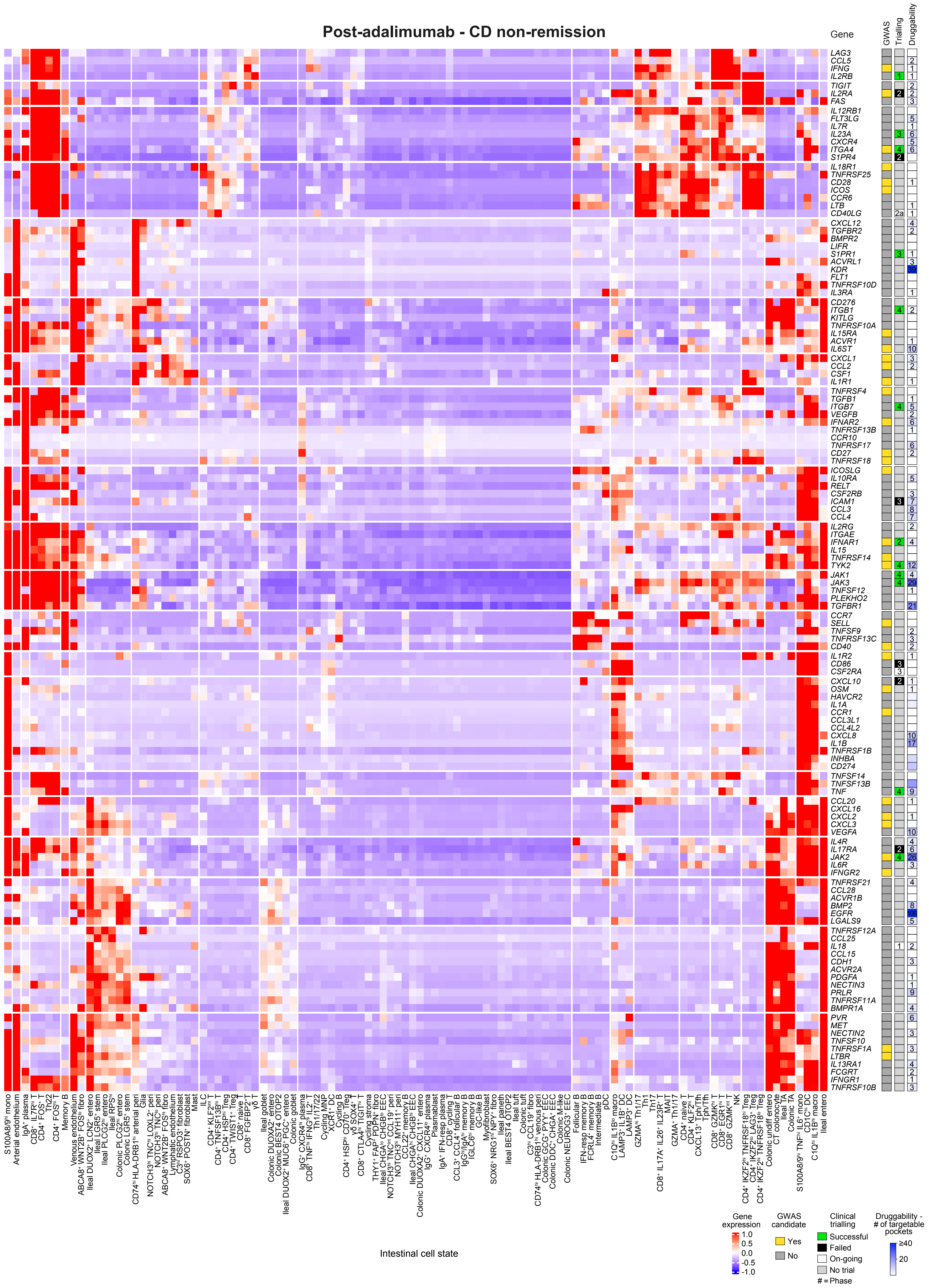
Post-adalimumab - CD non-remission
Gene
GWAS
Trialling
Druggability
LAG3
CCL5
IFNG
IL2RB
TIGIT
IL2RA
FAS
IL12RB1
FLT3LG
IL7R
IL23A
CXCR4
ITGA4
S1PR4
IL18R1
TNFRSF25
CD28
ICOS
CCR6
LTB
CD40LG
CXCL12
TGFBR2
BMPR2
LIFR
S1PR1
ACVRL1
KDR
FLT1
TNFRSF10D
IL3RA
CD276
ITGB1
KITLG
TNFRSF10A
IL15RA
ACVR1
IL6ST
CXCL1
CCL2
CSF1
IL1R1
TNFRSF4
TGFB1
ITGB7
VEGFB
IFNAR2
TNFRSF13B
CCR10
TNFRSF17
CD27
TNFRSF18
ICOSLG
IL10RA
RELT
CSF2RB
ICAM1
CCL3
CCL4
IL2RG
ITGAE
IFNAR1
IL15
TNFRSF14
TYK2
JAK1
JAK3
TNFSF12
PLEKHO2
TGFBR1
CCR7
SELL
TNFSF9
TNFRSF13C
CD40
IL1R2
CD86
CSF2RA
CXCL10
OSM
HAVCR2
IL1A
CCR1
CCL3L1
CCL4L2
CXCL8
IL1B
TNFRSF1B
INHBA
CD274
TNFSF14
TNFSF13B
TNF
CCL20
CXCL16
CXCL2
CXCL3
VEGFA
IL4R
IL17RA
JAK2
IL6R
IFNGR2
TNFRSF21
CCL28
ACVR1B
BMP2
EGFR
LGALS9
TNFRSF12A
CCL25
IL18
CCL15
CDH1
ACVR2A
PDGFA
NECTIN3
PRLR
TNFRSF11A
BMPR1A
PVR
MET
NECTIN2
TNFSF10
TNFRSF1A
LTBR
IL13RA1
FCGRT
IFNGR1
TNFRSF10B
S100A8/9hi mono
Arterial endothelium
IgA+ plasma
CD8+ IL7Rhi T
CD4+ FOS+ T
Th22
CD4+ FOShi T
Memory B
Venous endothelium
ABCA8+ WNT2B+ FOShi fibro
Ileal DUOX2+ LCN2+ entero
Ileal LGR5+ stem
Ileal PLCG2hi entero
Ileal RPShi
Colonic PLCG2hi entero
Colonic LGR5+ stem
CD74hi HLA-DRB1hi arterial peri
Cilia
NOTCH3hi TNChi LOXL2+ peri
NOTCH3hi TNClo peri
ABCA8+ WNT2B+ FOS+ fibro
Lymphatic endothelium
C3hi RSPO3+ fibroblast
SOX6+ POSTN+ fibroblast
Mast
ILC
CD4+ KLF2hi T
CD4+ TNFSF13Bhi T
CD4+ HSPhi Treg
CD4+ TWIST1+ Treg
CD8+ naive T
CD8+ FGFBP2+ T
γδ T
Ileal goblet
Colonic DUOX2+ entero
Colonic BEST4 OTOP2
Ileal DUOX2+ MUC6+ PGC+ entero
Colonic goblet
IgG+ CXCR4hi plasma
CD8+ TNFhi IFNGhi IL2+ T
Th1/17/22
Cycling MNP
XCR1+ DC
Cycling B
CD4+ HSPhi CD70+ Treg
γδ SOX4+ T
CD8+ CTLA4hi TIGIThi T
Cycling stroma
THY1+ FAP+ PDPN+ fibro
Ileal CHGAlo CHGBlo EEC
NOTCH3hi TNCint CCL19+ peri
NOTCH3hi MYH11+ peri
CCL22+ memory B
Ileal CHGA+ CHGB+ EEC
Colonic DUOX2+ CXCL11+ entero
IgG+ CXCR4lo plasma
Plasmablast
IgA+ IFN-resp plasma
CD8+ cycling T
CCL3+ CCL4+ follicular B
IgGhi/IgAhi memory B
IGLC6hi memory B
GC-like B
Myofibroblast
SOX6+ NRG1hi NPY+ fibro
Ileal paneth
Ileal BEST4 OTOP2
Ileal tuft
Colonic tuft
C3hi CCL19+ fibro
CD74hi HLA-DRB1hi venous peri
Colonic GCG+ PYY+ EEC
Colonic DDC+ CHGA+ EEC
Colonic M-like
Colonic NEUROG3+ EEC
Follicular B
IFN-resp memory B
FCRL4+ memory B
Intermediate B
pDC
C1Qhi IL1Blo macro
LAMP3+ IL1B+ DC
LAMP3+ DC
GZMA+ Th1/17
Th1
Th17
CD8+ IL17A+ IL26+ IL23R+ T
MAIT
GZMA+ Th1/17
CD4+ naive T
CD4+ KLF2hi T
CXCL13+ Tph/Tfh
Tph/Tfh
CD8+ GZMKhi T
CD8+ EGR1hi T
CD8+ GZMKint T
NK
CD4+ IKZF2hi TNFRSF18hi Treg
CD4+ IKZF2lo LAG3+ Treg
CD4+ IKZF2hi TNFRSF18lo Treg
Colonic undiff entero
CT colonocyte
Ileal TA
Colonic TA
S100A8/9hi TNFhi IL6+ mono
CD1Chi DC
C1Qhi IL1Bhi macro
Ileal entero
Intestinal cell state
Gene expression
1.0
0.5
0.0
-0.5
-1.0
GWAS candidate
Yes
No
Clinical trialling
Successful
Failed
On-going
No trial
# = Phase
Druggability - # of targetable pockets
≥40
20

Supplementary Figure 3| Therapeutic atlas for CD.

Samples from the CD non-remission group following treatment with adalimumab were pseudobulked at the cell-state resolution. A list of therapeutically relevant genes including curated cytokine and receptors from KEGG (M9809)[1], members of the JAK family, checkpoint co-inhibitory and co-stimulatory molecules, and cell trafficking molecules was compiled. Genes with expression in over 97% of cells were kept. Column-wise, and row-wise k-means clustering was applied. The first column to the right of the genes indicates whether the gene has been implicated in genome-wide association studies (GWAS; yellow). The second column indicates stage of development of therapeutic agent associated with the gene (Phase 1/2/3/4), green colour indicative of trial success. The third column is indicative of the number of druggable pockets as outlined on Pi[2]. DC, dendritic cell; EEC, enteroendocrine cell; GC, germinal centre; hi, high; IFN-resp, interferon-responsive; ILC, innate lymphoid cell; lo, low; macro, macrophage; MAIT, mucosal-associated invariant T; MNP, mononuclear phagocyte; mono, monocyte; NK, natural killer cells; pDC, plasmacytoid dendritic cell; peri, pericyte; TA, transit-amplifying; Tfh, $CD4^+$ follicular helper T cell; Tph, $CD4^+$ peripheral helper T cell; Th, $CD4^+$ T helper cell; Treg, $CD4^+$ regulatory T cell.

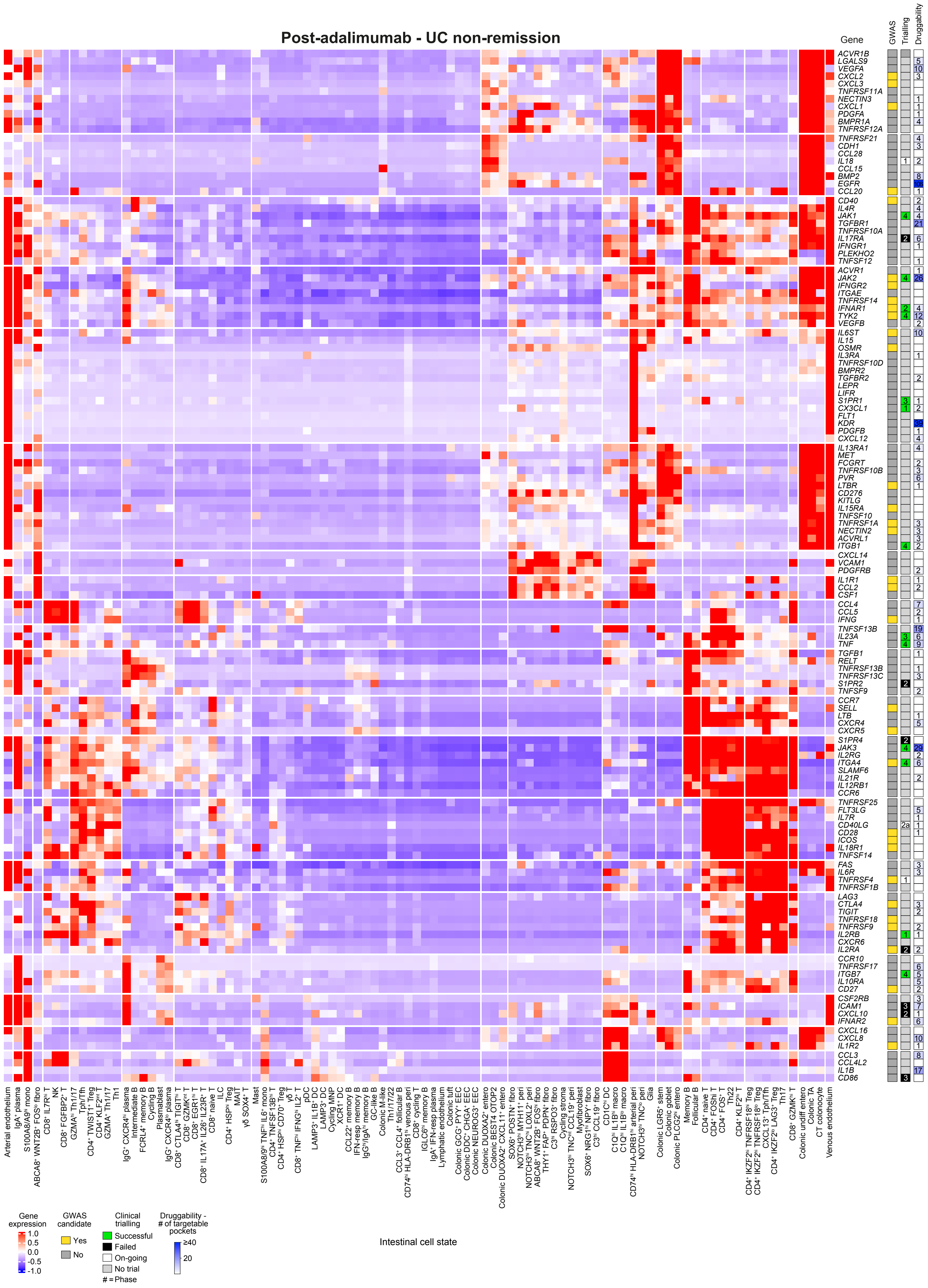
Post-adalimumab - UC non-remission
Gene
GWAS
Trialling
Druggability
ACVR1B
LGALS9
VEGFA
CXCL2
CXCL3
TNFRSF11A
NECTIN3
CXCL1
PDGFA
BMPR1A
TNFRSF12A
TNFRSF21
CDH1
CCL28
IL18
CCL15
BMP2
EGFR
CCL20
CD40
IL4R
JAK1
TGFBR1
TNFRSF10A
IL17RA
IFNGR1
PLEKHO2
TNFSF12
ACVR1
JAK2
IFNGR2
ITGAE
TNFRSF14
IFNAR1
TYK2
VEGFB
IL6ST
IL15
OSMR
IL3RA
TNFRSF10D
BMPR2
TGFBR2
LEPR
LIFR
S1PR1
CX3CL1
FLT1
KDR
PDGFB
CXCL12
IL13RA1
MET
FCGRT
TNFRSF10B
PVR
LTBR
CD276
KITLG
IL15RA
TNFSF10
TNFRSF1A
NECTIN2
ACVRL1
ITGB1
CXCL14
VCAM1
PDGFRB
IL1R1
CCL2
CSF1
CCL4
CCL5
IFNG
TNFSF13B
IL23A
TNF
TGFB1
RELT
TNFRSF13B
TNFRSF13C
S1PR2
TNFSF9
CCR7
SELL
LTB
CXCR4
CXCR5
S1PR4
JAK3
IL2RG
ITGA4
SLAMF6
IL21R
IL12RB1
CCR6
TNFRSF25
FLT3LG
IL7R
CD40LG
CD28
ICOS
IL18R1
TNFSF14
FAS
IL6R
TNFRSF4
TNFRSF1B
LAG3
CTLA4
TIGIT
TNFRSF18
TNFRSF9
IL2RB
CXCR6
IL2RA
CCR10
TNFRSF17
ITGB7
IL10RA
CD27
CSF2RB
ICAM1
CXCL10
IFNAR2
CXCL16
CXCL8
IL1R2
CCL3
CCL4L2
IL1B
CD86
Intestinal cell state
Gene expression
1.0
0.5
0.0
-0.5
-1.0
GWAS candidate
Yes
No
Clinical trialling
Successful
Failed
On-going
No trial
# = Phase
Druggability - # of targetable pockets
≥40
20

Supplementary Figure 4| Therapeutic atlas for UC.

Samples from the UC non-remission group following treatment with adalimumab were pseudobulked at the cell-state resolution. A list of therapeutically relevant genes: curated cytokine and receptors from KEGG (M9809)[1], members of the JAK family, checkpoint co-inhibitory and co-stimulatory molecules, cell trafficking molecules was compiled. Genes with expression in over 97% of cells were kept. Column-wise, and row-wise K-means clustering applied. The first column to the right of the genes indicates whether the gene has been implicated in in genome-wide association studies (GWAS; yellow). The second column indicates stage of development of therapeutic agent associated with the gene (Phase 1/2/3/4), green colour indicative of trial success. The third column is indicative of the number of druggable pockets as outlined on Pi[2]. DC, dendritic cell; EEC, enteroendocrine cell; GC, germinal centre; hi, high; IFN-resp, interferon-responsive; ILC, innate lymphoid cell; lo, low; macro, macrophage; MAIT, mucosal-associated invariant T; MNP, mononuclear phagocyte; mono, monocyte; NK, natural killer cells; pDC, plasmacytoid dendritic cell; peri, pericyte; TA, transit-amplifying; Tfh, $CD4^+$ follicular helper T cell; Tph, $CD4^+$ peripheral helper T cell; Th, $CD4^+$ T helper cell; Treg, $CD4^+$ regulatory T cell.

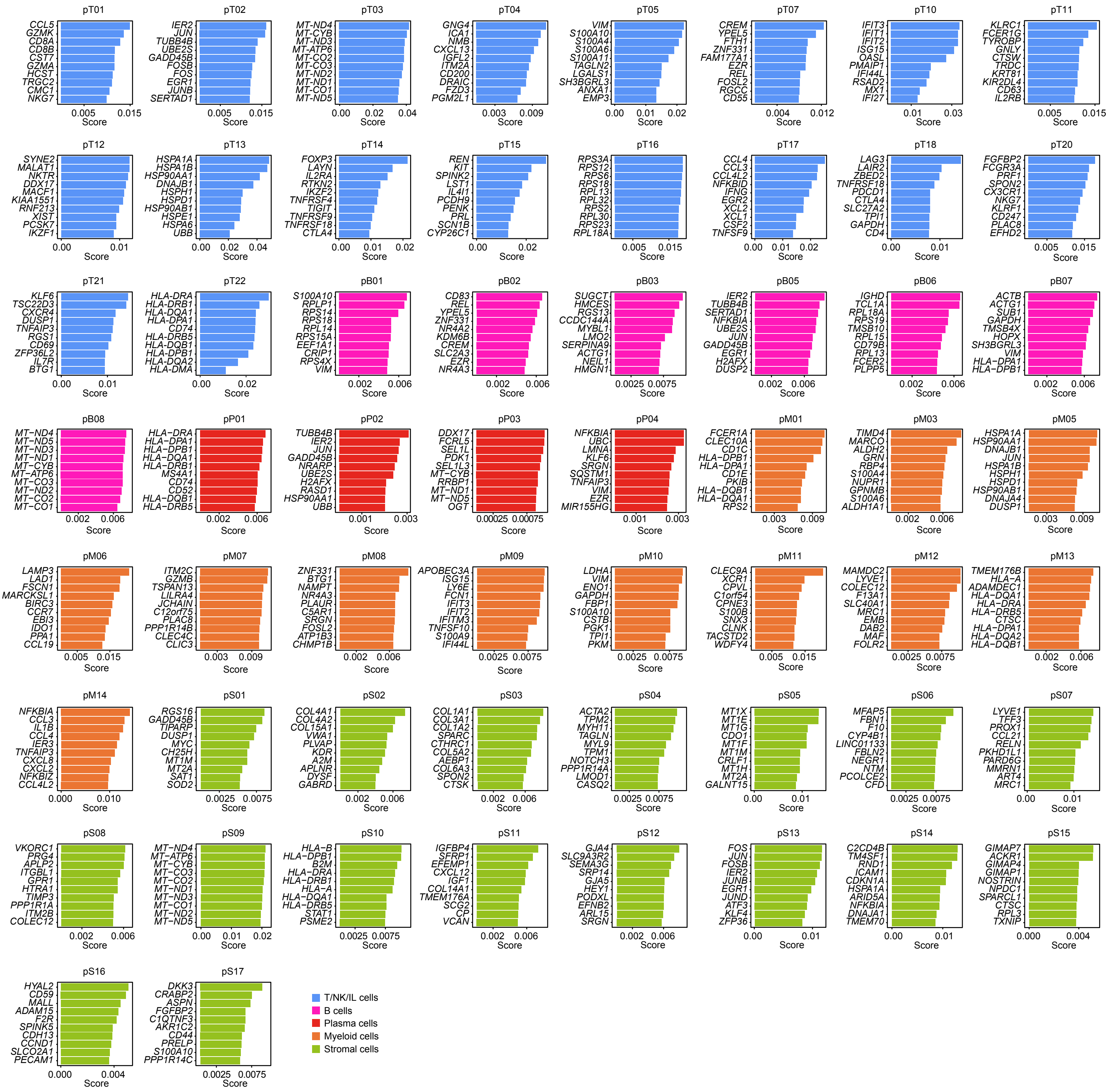

pT01
CCL5 GZMK CD8A CD8B CST7 GZMA HCST TRGC2 CMC1 NKG7
0.005 0.015 Score
pT02
IER2 JUN TUBB4B UBE2S GADD45B FOSB FOS EGR1 JUNB SERTAD1
0.005 0.015 Score
pT03
MT−ND4 MT−CYB MT−ND3 MT−ATP6 MT−CO2 MT−CO3 MT−ND2 MT−ND1 MT−CO1 MT−ND5
0.00 0.02 0.04 Score
pT04
GNG4 ICA1 NMB CXCL13 IGFL2 ITM2A CD200 DRAIC FZD3 PGM2L1
0.003 0.009 Score
pT05
VIM S100A10 S100A4 S100A6 S100A11 TAGLN2 LGALS1 SH3BGRL3 ANXA1 EMP3
0.00 0.01 0.02 Score
pT07
CREM YPEL5 FTH1 ZNF331 FAM177A1 EZR REL FOSL2 RGCC CD55
0.004 0.012 Score
pT10
IFIT3 IFIT1 IFIT2 ISG15 OASL PMAIP1 IFI44L RSAD2 MX1 IFI27
0.01 0.03 Score
pT11
KLRC1 FCER1G TYROBP GNLY CTSW TRDC KRT81 KIR2DL4 CD63 IL2RB
0.005 0.015 Score
pT12
SYNE2 MALAT1 NKTR DDX17 MACF1 KIAA1551 RNF213 XIST PCSK7 IKZF1
0.00 0.01 Score
pT13
HSPA1A HSPA1B HSP90AA1 DNAJB1 HSPH1 HSPD1 HSP90AB1 HSPE1 HSPA6 UBB
0.00 0.02 0.04 Score
pT14
FOXP3 LAYN IL2RA RTKN2 IKZF2 TNFRSF4 TIGIT TNFRSF9 TNFRSF18 CTLA4
0.00 0.01 0.02 Score
pT15
REN KIT SPINK2 LST1 IL4I1 PCDH9 PENK PRL SCN1B CYP26C1
0.00 0.02 Score
pT16
RPS3A RPS12 RPS6 RPS18 RPL13 RPL32 RPS2 RPL30 RPS23 RPL18A
0.005 0.015 Score
pT17
CCL4 CCL3 CCL4L2 NFKBID IFNG EGR2 XCL2 XCL1 CSF2 TNFSF9
0.00 0.01 0.02 Score
pT18
LAG3 LAIR2 ZBED2 TNFRSF18 PDCD1 CTLA4 SLC27A2 TPI1 GAPDH CD4
0.00 0.01 Score
pT20
FGFBP2 FCGR3A PRF1 SPON2 CX3CR1 NKG7 KLRF1 CD247 PLAC8 EFHD2
0.005 0.015 Score
pT21
KLF6 TSC22D3 CXCR4 DUSP1 TNFAIP3 RGS1 CD69 ZFP36L2 IL7R BTG1
0.00 0.01 Score
pT22
HLA−DRA HLA−DRB1 HLA−DQA1 HLA−DPA1 CD74 HLA−DRB5 HLA−DQB1 HLA−DPB1 HLA−DQA2 HLA−DMA
0.00 0.02 Score
pB01
S100A10 RPLP1 RPS14 RPS18 RPL14 RPS15A EEF1A1 CRIP1 RPS4X VIM
0.002 0.006 Score
pB02
CD83 REL YPEL5 ZNF331 NR4A2 KDM6B CREM SLC2A3 EZR NR4A3
0.002 0.006 Score
pB03
SUGCT HMCES RGS13 CCDC144A MYBL1 LMO2 SERPINA9 ACTG1 NEIL1 HMGN1
0.0025 0.0075 Score
pB05
IER2 TUBB4B SERTAD1 NFKBIA UBE2S JUN GADD45B EGR1 H2AFX DUSP2
0.002 0.006 Score
pB06
IGHD TCL1A RPL18A RPS19 TMSB10 RPL15 CD79B RPL13 FCER2 PLPP5
0.002 0.006 Score
pB07
ACTB ACTG1 SUB1 GAPDH TMSB4X HOPX SH3BGRL3 VIM HLA−DPA1 HLA−DPB1
0.002 0.006 Score
pB08
MT−ND4 MT−ND5 MT−ND3 MT−ND1 MT−CYB MT−ATP6 MT−CO3 MT−ND2 MT−CO2 MT−CO1
0.002 0.006 Score
pP01
HLA−DRA HLA−DPA1 HLA−DPB1 HLA−DRB1 HLA−DQA1 MS4A1 CD74 CD52 HLA−DQB1 HLA−DRB5
0.002 0.006 Score
pP02
TUBB4B IER2 JUN GADD45B NRARP UBE2S H2AFX RASD1 HSP90AA1 UBB
0.001 0.003 Score
pP03
DDX17 FCRL5 SEL1L PDK1 SEL1L3 MT−CYB RRBP1 MT−ND1 MT−ND5 OGT
0.00025 0.00075 Score
pP04
NFKBIA UBC LMNA KLF6 SRGN SQSTM1 TNFAIP3 VIM EZR MIR155HG
0.001 0.003 Score
pM01
FCER1A CLEC10A CD1C HLA−DPB1 HLA−DPA1 CD1E PKIB HLA−DQB1 HLA−DQA1 RPS2
0.003 0.009 Score
pM03
TIMD4 MARCO ALDH2 GRN RBP4 S100A4 NUPR1 GPNMB S100A6 ALDH1A1
0.002 0.006 Score
pM05
HSPA1A HSP90AA1 DNAJB1 JUN HSPA1B HSPH1 HSPD1 HSP90AB1 DNAJA4 DUSP1
0.003 0.009 Score
pM06
LAMP3 LAD1 FSCN1 MARCKSL1 BIRC3 CCR7 EBI3 IDO1 PPA1 CCL19
0.005 0.015 Score
pM07
ITM2C GZMB TSPAN13 LILRA4 JCHAIN C12orf75 PLAC8 PPP1R14B CLEC4C CLIC3
0.003 0.009 Score
pM08
ZNF331 BTG1 NAMPT NR4A3 PLAUR C5AR1 SRGN FOSL2 ATP1B3 CHMP1B
0.002 0.006 Score
pM09
APOBEC3A ISG15 LY6E FCN1 IFIT3 IFIT2 IFITM3 TNFSF10 S100A9 IFI44L
0.0025 0.0075 Score
pM10
LDHA VIM ENO1 GAPDH FBP1 S100A10 CSTB PGK1 TPI1 PKM
0.0025 0.0075 Score
pM11
CLEC9A XCR1 CPVL C1orf54 CPNE3 S100B SNX3 CLNK TACSTD2 WDFY4
0.005 0.015 Score
pM12
MAMDC2 LYVE1 COLEC12 F13A1 SLC40A1 MRC1 EMB DAB2 MAF FOLR2
0.0025 0.0075 Score
pM13
TMEM176B HLA−A ADAMDEC1 HLA−DQA1 HLA−DRA HLA−DRB5 CTSC HLA−DPA1 HLA−DQA2 HLA−DQB1
0.002 0.006 Score
pM14
NFKBIA CCL3 IL1B CCL4 IER3 TNFAIP3 CXCL8 CXCL2 NFKBIZ CCL4L2
0.000 0.010 Score
pS01
RGS16 GADD45B TIPARP DUSP1 MYC CH25H MT1M MT2A SAT1 SOD2
0.0025 0.0075 Score
pS02
COL4A1 COL4A2 COL15A1 VWA1 PLVAP KDR A2M APLNR DYSF GABRD
0.002 0.006 Score
pS03
COL1A1 COL3A1 COL1A2 SPARC CTHRC1 COL5A2 AEBP1 COL6A3 SPON2 CTSK
0.002 0.006 Score
pS04
ACTA2 TPM2 MYH11 TAGLN MYL9 TPM1 NOTCH3 PPP1R14A LMOD1 CASQ2
0.0025 0.0075 Score
pS05
MT1X MT1E MT1G CDO1 MT1F MT1M CRLF1 MT1H MT2A GALNT15
0.00 0.01 Score
pS06
MFAP5 FBN1 F10 CYP4B1 LINC01133 FBLN2 NEGR1 NTM PCOLCE2 CFD
0.0025 0.0075 Score
pS07
LYVE1 TFF3 PROX1 CCL21 RELN PKHD1L1 PARD6G MMRN1 ART4 MRC1
0.00 0.01 Score
pS08
VKORC1 PRG4 APLP2 ITGBL1 GPR1 HTRA1 TIMP3 PPP1R1A ITM2B COLEC12
0.002 0.006 Score
pS09
MT−ND4 MT−ATP6 MT−CYB MT−CO3 MT−CO2 MT−ND1 MT−ND3 MT−CO1 MT−ND2 MT−ND5
0.00 0.01 0.02 Score
pS10
HLA−B HLA−DPB1 B2M HLA−DRA HLA−DRB1 HLA−A HLA−DQA1 HLA−DRB5 STAT1 PSME2
0.0025 0.0075 Score
pS11
IGFBP4 SFRP1 EFEMP1 CXCL12 IGF1 CXCL14A1 TMEM176A SCG2 CP VCAN
0.002 0.006 Score
pS12
GJA4 SLC9A3R2 SEMA3G SRP14 GJA5 HEY1 PODXL EFNB2 ARL15 SRGN
0.002 0.006 Score
pS13
FOS JUN FOSB IER2 JUNB EGR1 JUND ATF3 KLF4 ZFP36
0.00 0.01 Score
pS14
C2CD4B TM4SF1 RND1 ICAM1 CDKN1A HSPA1A ARID5A NFKBIA DNAJA1 TMEM70
0.00 0.01 Score
pS15
GIMAP7 ACKR1 GIMAP4 GIMAP1 NOSTRIN NPDC1 SPARCL1 CTSC RPL3 TXNIP
0.000 0.004 Score
pS16
HYAL2 CD59 MALL ADAM15 F2R SPINK5 CDH13 CCND1 SLCO2A1 PECAM1
0.000 0.004 Score
pS17
DKK3 CRABP2 ASPN FGFBP2 C1QTNF3 AKR1C2 CD44 PRELP S100A10 PPP1R14C
0.0025 0.0075 Score
T/NK/IL cells
B cells
Plasma cells
Myeloid cells
Stromal cells

Supplementary Figure 5| Top weighted genes of GEPs in the synovium.
Weighted genes for each gene expression programme (GEP) derived from the synovium. cNMF was run separately in: T cells, B cells, plasma cells, myeloid cells and stromal cells. See **Supplementary Table 9** for full list of weighted genes, as well as results of overrepresentation analysis. See **Supplementary Table 9** for results of enrichment testing of GEPs in inflammation. pB, B cell GEP; pM, myeloid cell GEP; pP, plasma cell GEP; pS, stromal cell GEP; pT, T/NK cell GEP.

References:

1. Kanehisa, M. *et al*. KEGG as a reference resource for gene and protein annotation. *Nucleic Acids Res.* **44**, D457–462 (2016).

2. Fang, H. & Knight, J.C. Priority index: database of genetic targets in immune-mediated disease. *Nucleic Acids Res*. **50**, D1358–1367 (2022).
