# Supplementary material for: A longitudinal single-cell atlas of anti-tumour necrosis factor treatment in inflammatory bowel disease
Source: Nat Immunol. 2024 Oct 22;25(11):2152–65. doi: 10.1038/s41590-024-01994-8 (PMC11519010; doi:10.1038/s41590-024-01994-8)
Supplement: Supplementary file 2 — Reporting Summary [file 41590_2024_1994_MOESM2_ESM.pdf]

Corresponding author(s): Christopher Buckley
Last updated by author(s): Sep 16, 2024

Reporting Summary

Nature Portfolio wishes to improve the reproducibility of the work that we publish. This form provides structure for consistency and transparency in reporting. For further information on Nature Portfolio policies, see our Editorial Policies and the Editorial Policy Checklist.

Statistics

For all statistical analyses, confirm that the following items are present in the figure legend, table legend, main text, or Methods section.

n/a	Confirmed	
☐	☒	The exact sample size (n) for each experimental group/condition, given as a discrete number and unit of measurement
☐	☒	A statement on whether measurements were taken from distinct samples or whether the same sample was measured repeatedly
☐	☒	The statistical test(s) used AND whether they are one- or two-sided<br>*Only common tests should be described solely by name; describe more complex techniques in the Methods section.*
☐	☒	A description of all covariates tested
☐	☒	A description of any assumptions or corrections, such as tests of normality and adjustment for multiple comparisons
☐	☒	A full description of the statistical parameters including central tendency (e.g. means) or other basic estimates (e.g. regression coefficient) AND variation (e.g. standard deviation) or associated estimates of uncertainty (e.g. confidence intervals)
☐	☒	For null hypothesis testing, the test statistic (e.g. F, t, r) with confidence intervals, effect sizes, degrees of freedom and P value noted<br>*Give P values as exact values whenever suitable.*
☒	☐	For Bayesian analysis, information on the choice of priors and Markov chain Monte Carlo settings
☒	☐	For hierarchical and complex designs, identification of the appropriate level for tests and full reporting of outcomes
☐	☒	Estimates of effect sizes (e.g. Cohen's d, Pearson's r), indicating how they were calculated

Our web collection on statistics for biologists contains articles on many of the points above.

Software and code

Policy information about availability of computer code

Data collection	Single cell RNA sequencing, Cell DIVE Multiplex Imaging
Data analysis	All code and associated information used to generate results will be available (github.com/DendrouLab/TAURUS_paper/) upon acceptance of the paper. No new algorithms were generated in this work. Python 3.7.4 and R 3.6.2 were used. Cell Ranger v3.1.0 was used to process scRNAseq data. Panpipes (https://github.com/Dendroulab/panpipes) v0.1 was used for downstream processing including quality control, doublet removal (scrublet v0.2.1), identifying highly variable genes and clustering. Differential abundance was conducted using MASC(v0.1.0). Differential expression tools used during the course of analysis include: limma (v3.46.0), DESeq2 (v1.30.1) and MAST (1.22.0). escape (v1.1.1) was used to generate inflammation scores for pseudobulked samples. PROGENy-py (1.0.6) was used to derive scores for TNF signalling. cNMF (v1.3) was used to generate gene expression profiles (https://github.com/dylkot/cNMF). Community detection within the network of significant correlations was performed using https://github.com/pouyaesm/signed-community-detection (v1.1.1). lmerTest (v3.1.3) package was used to assess TNF signalling before and after treatment, and test for enrichment of gene expression programs in inflammation. scikit-learn (v1.1.1) was used for checking scRNA-seq derived GEP profiles in bulk RNA sequencing data. FlowJo v10 was used.

For manuscripts utilizing custom algorithms or software that are central to the research but not yet described in published literature, software must be made available to editors and reviewers. We strongly encourage code deposition in a community repository (e.g. GitHub). See the Nature Portfolio guidelines for submitting code & software for further information.

Data

Policy information about availability of data

All manuscripts must include a data availability statement. This statement should provide the following information, where applicable:

- Accession codes, unique identifiers, or web links for publicly available datasets
- A description of any restrictions on data availability
- For clinical datasets or third party data, please ensure that the statement adheres to our policy

All data (de-identified) will be uploaded to GEO (raw and processed scRNAseq data), and Zenodo (10.5281/zenodo.13768607). A link to a web platform for interactive browsing will be available on Zenodo. Publicly available data used for analysis was downloaded from GEO (GSE16879) and E-MTAB-11611 (R4RA). Human transcriptome GRCh38-3.0.0 reference available at: https:// www.10xgenomics.com/support/software/cell-ranger/downloads/cr-ref-build-steps/.

Research involving human participants, their data, or biological material

Policy information about studies with human participants or human data. See also policy information about sex, gender (identity/presentation), and sexual orientation and race, ethnicity and racism.

Reporting on sex and gender	Both male and female sexes are reported in the data. Information pertaining to this was collected from electronic patient records. Cohort of 41 patients comprised of 19 males and 22 females.
Reporting on race, ethnicity, or other socially relevant groupings	Ethnicity has been reported in the metadata.
Population characteristics	Biologic naive patients were recruited. Patient cohort consisted of patients with CD (n=16), UC (n=22), and health controls (n=3). Mean age in years (SD), was 36 (10.6) for CD, 33 (10.10) for UC, and 66(3.68) for healthy controls. Mean disease duration was 96 months (76) and 73 (71) for CD and UC respectively. Montreal classifications, smoking data, and follow up data is reported in Supplementary Table 1.
Recruitment	Biologic-naive patients with IBD to be escalated to adalimumab were recruited from the IBD outpatient clinic at the John Radcliffe Hospital in Oxford, UK. Patients with clinically diagnosed RA were recruited to and followed up in an observational standard of care cohort in Birmingham, UK. A potential self-selection bias is whether certain patients are more likely to participate in research. Our cohort was well-represented for inflammation severity, and remission status outcomes did not differ by inflammation severity at baseline.
Ethics oversight	IBD cohort for the paper was recruited under: [(IBD Cohort 09/H1204/30)/(GI Ethics 16/YH/0247)] provided by Yorkshire & The Humber - Sheffield Research Ethics Committee. RA cohort were recruited under: [South Birmingham Research Ethics Committee: 14/WM/1109) (West Midlands Black Country Research Ethics Committee: 07 /H1203/57)].

Note that full information on the approval of the study protocol must also be provided in the manuscript.

Field-specific reporting

Please select the one below that is the best fit for your research. If you are not sure, read the appropriate sections before making your selection.

☒ Life sciences ☐ Behavioural & social sciences ☐ Ecological, evolutionary & environmental sciences

For a reference copy of the document with all sections, see nature.com/documents/nr-reporting-summary-flat.pdf

Life sciences study design

All studies must disclose on these points even when the disclosure is negative.

Sample size	This was an observational study. The number of available samples was dictated by the number of patients being escalated to adalimumab, as well as patient willingness to undergo endoscopy, as well as patient welfare at the point of sample collection. Conclusions in the manuscript relating to this data are supported by appropriate statistical tests, and where possible all datapoints are shown.
Data exclusions	Exclusion from study occurred if the patient developed antibodies to adalimumab sufficient to result in undetectable drug levels. This was a pre-set exclusion criteria.
Replication	Given the longitudinal element of recruitment, and the nature of these samples (collected in addition to routine clinical samples, and cost), further samples are difficult to come by. Patient data was analysed at a cohort level, using the relevant patients/samples/time points to derive statistically meaningful conclusions.
Randomization	This was an observation cohort with only one experimental group, hence randomization was not relevant to this study.
Blinding	All human samples were anonymised before data collection by giving them a unique ID number. This ID was used during analysis to blind investigators. Histopathology scoring was also performed in a blinded fashion.

Reporting for specific materials, systems and methods

We require information from authors about some types of materials, experimental systems and methods used in many studies. Here, indicate whether each material, system or method listed is relevant to your study. If you are not sure if a list item applies to your research, read the appropriate section before selecting a response.

Materials & experimental systems

n/a	Involved in the study
☐	☒ Antibodies
☒	☐ Eukaryotic cell lines
☒	☐ Palaeontology and archaeology
☒	☐ Animals and other organisms
☒	☐ Clinical data
☒	☐ Dual use research of concern
☒	☐ Plants

Methods

n/a	Involved in the study
☒	☐ ChIP-seq
☐	☒ Flow cytometry
☒	☐ MRI-based neuroimaging

Antibodies

Antibodies used

Details provided in the manuscript. Multiplexed imaging using the CellDive consisted of staining using commercially available antibodies. The following antibodies were stained for: antigen (Clone), company (Catalog number), Conjugation (Lot – Concentration)

CD68 (EPR20545), Abcam (ab280860) AlexaFluor555 (GR3379176-3 – 5ug/ml); CD3 (SP162), Abcam (ab245731) AlexaFluor555 (GR3316803-2 – 5ug/ml);CCL19 (polyclonal goat), BioTechne (AF361) AlexaFluor 647 (BAU0819051 – 5ug/ml);CD8A (C8/144B), Biolegend (372906) AlexaFluor 647 (B247314 – 10ug/ml);CK8 (EP1628Y), Abcam (ab192467) AlexaFluor488 (GR3262903-1 – 5ug/ml);CD4 (EPR6855), Abcam (ab280849) AlexaFluor555 (GR3388856-2 – 5ug/ml); CXCL13 (polyclonal) Biotechne (AF801) AlexaFluor 555 (BAS0317111 – 10ug/ml); CD20 (EP459Y) Abcam (ab198941) Alexa Fluor 488 (GR3404776-4 – 5 ug/ml); CD208 (EPR24265-8) Abcam (ab281573) Alexa Fluor 555 (GR3386297-2 - 2.5ug/ml);CXCL9 (E6Z5W) Cell signalling (37438SF) Alexa Fluor 555 (1 – 5ug/ml); S100A9 (EPR35555) Abcam (ab271864) Alexa Fluor 555 (GR3430572-2 – 2.5ug/ml); Ki67 (SP6) Abcam (ab281847) Alexa Fluor 488 (GR3437226-8 – 5ug/ml); MPO (A-5) SantaCruz (sc-365436) Alex Fluor 546 (C2921 – 5ug/ml); Granzyme B (D6E9W) Cell signalling (79903SF) (2 – 3ug/ml); CD66B (G10F5) Biolegend (305110) Alexa Fluor 647 (B257493 – 2ug/ml); CD14 (EPR3653) Abcam (ab226121) Alexa Fluor 647 (GR3386297-2 – 5ug/ml); CCR7 (EPR23192-57) Abcam (ab275165) Alexa Fluor 647 (GR3369283-2 – 5ug/ml); CD11c (EP1347Y) Abcam (ab279329) Alexa Fluor 555 (GR3370220-1 – 5ug/ml); CD40 (D8W3N) Cell signalling (77841SF) Alexa Fluor 555 (lot 1 – 5ug/ml); PD1 (NAT105) Abcam (ab220301) Alexa Fluor 647 (GR3365990-1 – 5ug/ml); MZB1 (22) Novus (NBP2-90320) (D124248 - 5ug/ml); COL1A1 (EPR7785) Abcam (ab275996) (GR3419821-6 - 5ug/ml).

For flow cytometry on PBMCs, the following antibodies were used: mouse anti-human TNFR1-APC mAb (clone W15099A, BioLegend, lot: B363511, 2µg/ml]; rat anti-human TNFR2-PE mAb [clone hTNFR-M1, BD BioSciences, lot: B363682, 2µg/ml]; mouse anti-human TNF-alpha [clone Mab11, BioLegend, lot: B375242, 2µg/ml].

Validation

Antibodies from Abcam and Cell Signalling have been validated for IHC-P and tested to react with human by manufacturer. CXCL13 and CCL19 antibodies were tested on human tonsil FFPE section.

Plants

Seed stocks

n/a

Novel plant genotypes

n/a

Authentication

n/a

Flow Cytometry

Plots

Confirm that:

☒ The axis labels state the marker and fluorochrome used (e.g. CD4-FITC).

☒ The axis scales are clearly visible. Include numbers along axes only for bottom left plot of group (a 'group' is an analysis of identical markers).

☒ All plots are contour plots with outliers or pseudocolor plots.

☒ A numerical value for number of cells or percentage (with statistics) is provided.

Methodology

Sample preparation	Single-cell suspensions derived from PBMCs were stained for 20 minutes on ice in buffer solution (PBS, 0.1% BSA, 5mM EDTA) containing antibodies at 2µg/ml. Cells were then centrifuged at 300xg for 5 minutes (4C) and washed twice in buffer. Cells were then either fixed for 20 minutes in 4% paraformaldehyde (RT), or fixed/permeabilised according to the manufacturer's instruction (BD BioSciences Cytofix/Cytoperm, #554714); for intracellular staining, antibodies were incubated in permeabilisation buffer for 30 minutes (RT). Stained cells were acquired on a BD LSRII.
Instrument	BD LSRII
Software	FlowJo v10
Cell population abundance	Out of live CD45+ cells, the subsets analysed had a frequency of at least 5%.
Gating strategy	Enclosed in Extended Data Fig. 5. Cells were gated on FSC and SSC to exclude debris. Then, single cells were gated using FSC-A and FSC-H. Gating was performed to isolate the live CD45+ cells. CD3 and CD19 markers were used to identify T and B cells. CD3- and CD19- were gated based on CD14 and SSC to identify mononuclear phagocytes and granulocytes.

☒ Tick this box to confirm that a figure exemplifying the gating strategy is provided in the Supplementary Information.
